# Mer regulates microglial/macrophage M1/M2 polarization and alleviates neuroinflammation following traumatic brain injury

**DOI:** 10.1186/s12974-020-02041-7

**Published:** 2021-01-05

**Authors:** Haijian Wu, Jingwei Zheng, Shenbin Xu, Yuanjian Fang, Yingxi Wu, Jianxiong Zeng, Anwen Shao, Ligen Shi, Jianan Lu, Shuhao Mei, Xiaoyu Wang, Xinying Guo, Yirong Wang, Zhen Zhao, Jianmin Zhang

**Affiliations:** 1 Department of Neurosurgery, Second Affiliated Hospital, School of Medicine, Zhejiang University, 88 Jiefang Road, Hangzhou, 310009 Zhejiang China; 2 Department of Neurosurgery, Sir Run Run Shaw Hospital, School of Medicine, Zhejiang University, Hangzhou, Zhejiang China; 3 Center for Neurodegeneration and Regeneration, Zilkha Neurogenetic Institute and Department of Physiology and Biophysics, Keck School of Medicine, University of Southern California, 1501 San Pablo Street, Los Angeles, CA 90089 USA; 4 Brain Research Institute, Zhejiang University, Hangzhou, China; 5 Collaborative Innovation Center for Brain Science, Zhejiang University, Hangzhou, China

**Keywords:** Mer, Microglia/macrophage, M1/M2 polarization, Neuroinflammation, TBI

## Abstract

**Background:**

Traumatic brain injury (TBI) is a leading cause of death and disability worldwide. Microglial/macrophage activation and neuroinflammation are key cellular events following TBI, but the regulatory and functional mechanisms are still not well understood. Myeloid-epithelial-reproductive tyrosine kinase (Mer), a member of the Tyro-Axl-Mer (TAM) family of receptor tyrosine kinases, regulates multiple features of microglial/macrophage physiology. However, its function in regulating the innate immune response and microglial/macrophage M1/M2 polarization in TBI has not been addressed. The present study aimed to evaluate the role of Mer in regulating microglial/macrophage M1/M2 polarization and neuroinflammation following TBI.

**Methods:**

The controlled cortical impact (CCI) mouse model was employed. Mer siRNA was intracerebroventricularly administered, and recombinant protein S (PS) was intravenously applied for intervention. The neurobehavioral assessments, RT-PCR, Western blot, magnetic-activated cell sorting, immunohistochemistry and confocal microscopy analysis, Nissl and Fluoro-Jade B staining, brain water content measurement, and contusion volume assessment were performed.

**Results:**

Mer is upregulated and regulates microglial/macrophage M1/M2 polarization and neuroinflammation in the acute stage of TBI. Mechanistically, Mer activates the signal transducer and activator of transcription 1 (STAT1)/suppressor of cytokine signaling 1/3 (SOCS1/3) pathway. Inhibition of Mer markedly decreases microglial/macrophage M2-like polarization while increases M1-like polarization, which exacerbates the secondary brain damage and sensorimotor deficits after TBI. Recombinant PS exerts beneficial effects in TBI mice through Mer activation.

**Conclusions:**

Mer is an important regulator of microglial/macrophage M1/M2 polarization and neuroinflammation, and may be considered as a potential target for therapeutic intervention in TBI.

**Supplementary Information:**

The online version contains supplementary material available at 10.1186/s12974-020-02041-7.

## Background

Traumatic brain injury (TBI) is a devastating neurological disease and a major cause of death and disability worldwide, not only young people but also the elderly [1, 2]. It is estimated that approximately 70 million individuals suffer a TBI annually [3]. Notably, the pathophysiological processes of TBI are rather complex, which occur in the acute phase and yet continue to evolve over time, leading to persistent, sometimes life-long, consequences [4]. TBI is an important risk factor for a variety of chronic neurological disorders, such as epilepsy, stroke, psychiatric illness, and neurodegenerative diseases [5]. There is no doubt that TBI not only severely affects the daily life of survivors and their families, but also results in substantial socio-economic impacts [6]. However, a comprehensive understanding of this notorious disease is still lacking, and the need for therapeutics that effectively improve long-term functional outcomes in TBI survivors remains unmet.

Mechanistically, both primary and secondary injury mechanisms are involved in the neuropathology of TBI. Secondary brain injury, which occurs from initial insults to the brain, is an important determinant of TBI outcomes [7]. It consists of ionic homeostasis disturbance, excitotoxicity, oxidative stress, inflammation, and cell death, among others. In particular, neuroinflammation is one of the most common and important cellular events following TBI [8]. It can evolve over minutes to days, months, and even years after the initial injury and contributes to the development of both acute and chronic neurological consequences post-insult [9, 10]. Microglia, the major resident immune cells in the brain, are presumed to be key players in neuroinflammation after TBI [11]. Also, peripherally derived macrophages are actively involved in acute neuroinflammatory responses after TBI [12]. It has reported that microglia/macrophages are highly plastic and assume their phenotypes dependent on different microenvironmental cues, such as pro-inflammation and anti-inflammation, among others [13]. The pro-inflammatory M1-like phenotype expressing signature markers such as CD16 and CD32 tends to release destructive mediators, including tumor necrosis factor-α (TNF-α), interleukin-1β (IL-1β), and inducible nitric oxide synthase (iNOS). In contrast, the anti-inflammatory M2-like phenotype, characterized by the molecular signatures of CD206 and arginase 1 (Arg-1), produces beneficial mediators such as interleukin-10 (IL-10) and transforming growth factor-β (TGF-β) [14]. Notably, the M1-like phenotype is more likely associated with uncontrolled neuroinflammation often observed in neurodegenerative diseases, while the M2-like phenotype promotes inflammation resolution and tissue repair [15]. Thus, a balanced response between polarized microglial/macrophage phenotypes is essential for immune homeostasis in the brain.

Currently, the dualistic roles of distinctly polarized microglial/macrophage populations have been demonstrated in major central nervous system diseases (CNS), including TBI [16, 17]. Studies from animal models indicated that despite both M1-like and M2-like polarized microglial/macrophage being activated after TBI, the activity of M2-like response declines over time, whereas the pathological M1-like effect can persist for a long period of time post-insult [16, 17], and modulating the M1-M2 polarization balance has been shown to be beneficial for functional outcomes [18, 19]. However, the molecular mechanisms underlying microglial/macrophage polarization in TBI are still not fully understood. Remarkably, the neuroinflammatory responses following TBI are highly complex and cannot be so easily delineated by using a binary M1/M2 nomenclature [20]. For instance, in a controlled cortical impact (CCI) mouse model of TBI, Morganti et al. found that activated microglia/macrophages adjacent to the ipsilateral perilesional cortical tissue simultaneously express both “M1-like” and “M2-like” phenotypic markers across multiple time points after injury [21]. Instead of switching to a polarized “M1-only” or “M2-only” phenotype, these cells shape their responses in complex and mixed phenotypes during CNS injury and disease, including TBI [12, 20, 21]. And the polarization responses of these cells are heterogeneous and overlapping after injury, such that pro-inflammatory responses are occurring concurrently with the expression of anti-inflammatory and tissue repair mediators [12].

Myeloid-epithelial-reproductive tyrosine kinase (Mer), a member of the Tyro-Axl-Mer (TAM) family of receptor tyrosine kinases [22], plays a critical role in the regulation of inflammatory responses in peripheral macrophages [23–26] and brain microglial cells [27–29]. Specifically, Mer signaling stires macrophage polarization towards an M2-like phenotype [30]. For instance, it reported that protein S (PS)-mediated activation of Mer inhibited M1-like polarization of peritoneal and tumor-derived macrophages [31]. Besides, growth arrest-specific protein 6 (Gas6)-mediated Mer signaling increases the expression of M2-associated genes such as *Arg-2* and *VEGF* in bone marrow-derived macrophages [32]. Mer signaling is also required for the efficient clearance of early apoptotic cells by M2c-like macrophages [33]. However, the function of Mer in regulating the innate immune response and microglial/macrophage M1/M2 polarization in TBI has not been addressed. In the present study, we hypothesized that Mer upregulation is a key modulator of microglial/macrophage M1/M2 polarization and neuroinflammation in the acute stage of TBI. We found that inhibition of Mer shifts microglial/macrophage polarization toward the M1-like phenotype via regulating the signal transducer and activator of transcription 1 (STAT1)/suppressor of cytokine signaling 1/3 (SOCS1/3) pathway, which exacerbates neuroinflammation and secondary brain damage following TBI. In contrast, activation of Mer signaling increases microglial/macrophage M2-like polarization while decreases M1-like polarization and confers neuroprotection after TBI.

## Materials and methods

### Animals

Adult male and female C57BL/6 mice (22–27 g, aged 8–10 weeks) which were purchased from Slac Laboratory Co., Ltd. (Shanghai, China) were used for this study. Notably, male mice were used in this study except that 14 female mice were tested to compare the effect of sex differences on Mer expression and neurofunctional outcomes after TBI. All mice were housed in filter-top cages and fed a standard diet, with a 12-h light/dark cycle. Free access to food and water as well as controlled temperature and humidity were provided. All animal experiments were performed according to the Institutional Animal Care and Use Committee of Zhejiang University. The procedures were conducted according to the National Institutes of Health’s Guide for the Care and the Use of Laboratory Animals and the ARRIVE (Animal Research: Reporting In Vivo Experiments) guidelines. All efforts were made to minimize animal suffering and the number of animals sacrificed.

### Randomization and blinding

All animals were randomized for group allocation and surgical procedures and included in the analysis. The operators responsible for the experimental procedures and data analysis were blinded and unaware of group allocation throughout the experiments.

### TBI model

TBI was induced in C57BL/6 mice using a CCI model (Suppl. Fig. 1A-C), as described previously [34]. Briefly, mice were anesthetized by an intraperitoneal injection of pentobarbital sodium (50 mg/kg) and placed in the stereotaxic frame. A 4-mm-diameter craniotomy was performed using a portable drill over the right parietal cortex between bregma and lambda, 2 mm lateral to the midline. The dura mater was kept intact over the cortex. The CCI was performed perpendicular to the brain surface using a PinPoint™ Precision Cortical Impactor (Cary, NC, USA) with a 3-mm-diameter impact tip. The impact velocity is 3 m/s, the impact duration is 150 ms, and the impact depth is 2 mm. After TBI, the bone flap was immediately replaced and sealed, and the scalp was sutured closed. The animal’s core body temperature was maintained at 37 ± 0.5 °C with a thermostatically controlled heating pad during surgery. Sham animals were subjected to all aspects of the protocol (surgery, anesthesia, craniotomy, injection, and recovery) except for CCI. Recombinant PS (0.2 mg/kg) (9489-PS, R&D Systems) was administered via the tail vein at 1 h, 1 day, and 2 days after the CCI [35, 36] (Suppl. Fig. 2).

### Intracerebroventricular (i.c.v.) injection

Previous studies demonstrated that *i.c.v.* delivery of siRNA can efficiently silence target gene expression in the brain with a range of 50–80% [37–39]. The *i.c.v.* administration of siRNA was performed as previously described [40] (Suppl. Fig. 1D-F). A 1-mm-diameter burr hole was drilled into the right side of the skull (1.0 mm lateral and − 0.25 mm anterior-posterior (AP) to bregma). Mer siRNA or scrambled siRNA (500 pmol, Thermo Fisher Scientific) mixed with the transfection reagent (Engreen Biosystem Co., Ltd.), for a total volume of 2 μl, was delivered into the ipsilateral ventricle (1.0 mm lateral and − 0.25 mm AP to bregma, 2.5 mm dorsoventral (DV) below the skull). The injection was performed at a rate of 0.5 μl/min, then the needle stayed in the brain for another 5 min after injection to avert leakage, and then the burr hole was sealed with bone wax, and the incision was closed with sutures. Mice were placed in individual recovery cages. The *i.c.v.* injection of siRNA was performed 1 day before and 10 min after TBI.

### Neurobehavioral function assessment

#### Modified neurological severity scores (mNSS)

The mNSS score was assessed as previously reported [34]. It includes sensory, motor, balance, and reflex tests. The neurological function was graded on a scale of 0–18 (normal score, 0; maximal deficit score, 18) and recorded before TBI, as well as at 1, 3, and 7 days after TBI.

#### Foot-fault test

It was performed to evaluate the motor function similarly to that described previously [40]. Mice were placed on hexagonal grids of different sizes. Mice placed their paws on the wire while moving along the grid. With each weight-bearing step, the paw may fall or slip between the wire. This was recorded as a foot fault. The total number of steps (movement of each forelimb) that the mouse used to cross the grid was counted, and the total number of foot faults for each forelimb was recorded. The percentage of forelimb faults to the total number of steps was calculated.

#### Rotarod test

It was performed to test the motor coordination and the limb strength as previously described with the Rota-Rod Treadmills (BW-ZH600, Shanghai Bio-will Co., Ltd.) [40]. Test sessions consist of six trials at a variable speed (an initial velocity of 5 rpm was used for the first 10 s, a linear increase from 5 to 10 rpm for the next 30 s, and a linear increase from 10 to 20 rpm between 40 and 90 s). The final score was determined as the mean time that a mouse was able to remain on the rod over six trials.

### Brain water content measurement

Brain water content was measured as previously reported [34]. Briefly, mice were sacrificed 72 h after TBI, and the brains were obtained without transcardiac perfusion. Tissue samples of injured hemispheres were dissected and weighed on an electric analytic balance to obtain the wet weight and then dried at 100 °C for 48 h to obtain the dry weight. Brain water content was calculated using the following formula: brain water content (%) = (wet weight − dry weight)/wet weight × 100%.

### Contusion volume assessment

To measure the contusion volume in the ipsilateral cortex 72 h after TBI, cresyl violet-stained sections were digitized and analyzed by using ImageJ (National Institutes of Health, Bethesda, MD, USA) as previously reported [41]. The volume was computed by adding the injury areas and multiplying with the inter-slice distance (500 μm). Hemispheric tissue loss was expressed as a percentage that was calculated by the use of the following formulae: [(contralateral hemispheric volume − ipsilateral hemispheric volume)/(contralateral hemispheric volume) × 100%].

### Quantitative RT-PCR

Total RNA was isolated from sham and injured brains using the TRIzol reagent (Sigma-Aldrich, St. Louis, MO, USA) according to the manufacturer’s instructions. Then, RNA (1 μg) from each sample was reverse-transcribed to cDNA by PrimeScript^TM^ RT reagent kit (Takara Bio Inc, Shiga, Japan). Afterward, quantitative reverse transcription-polymerase chain reaction (RT-PCR) was conducted with SYBR® Premix Ex Taq™ (Takara Bio Inc, Shiga, Japan) on a 7300 Plus Read-Time PCR System (Thermo Fisher Scientific). The cDNA was used as a template in a 20 μl reaction volume (10 μl of PCR mix, 5 pmol of forward and reverse primers, 1 μl cDNA template and proper volume of water). The PCR reaction was performed as follows: Cycling conditions began with an initial DNA denaturation step at 95 °C for 20 s, followed by 40 cycles at 94 °C for 15 s, 56 °C for 30 s, and 72 °C for 25 s. Each sample was examined in triplicate. The threshold cycle (CT) readings were collected, and the relative expression of mRNA of target genes was calculated with the 2^−ΔΔCT^ method and was normalized to the glyceraldehyde 3-phosphate dehydrogenase (GAPDH) level in all samples. The expression levels of the mRNA were then reported as fold changes versus sham controls. The sequences of the primer pairs for target genes are as shown below:

**Table:** 

Gene	Forward primer sequence (5′–3′)	Reverse primer sequence (5′–3′)
*CD16*	TTTGGACACCCAGATGTTTCAG	GTCTTCCTTGAGCACCTGGATC
*CD32*	AATCCTGCCGTTCCTACTGATC	GTGTCACCGTGTCTTCCTTGAG
*iNOS*	CAAGCACCTTGGAAGAGGAG	AAGGCCAAACACAGCATACC
*CD206*	CAAGGAAGGTTGGCATTTGT	CCTTTCAGTCCTTTGCAAGC
*Arg-1*	TCACCTGAGCTTTGATGTCG	CTGAAAGGAGCCCTGTCTTG
*IL-10*	CCAAGCCTTATCGGAAATGA	TTTTCACAGGGGAGAAATCG
*Mer*	CCTCTGCTTCGCCACATCTGTATG	GACCAGCCAATCTCATTCCGACAG
*SOCS-1*	CCTCGTCCTCGTCTTCGTCCTC	GAAGGTGCGGAAGTGAGTGTCG
*SOCS-3*	GACCAAGAACCTACGCATCCAGTG	GCACCAGCTTGAGTACACAGTCG
*GAPDH*	TTCAACGGCACAGTCAAGG	CACCAGTGGATGCAGGGAT

### Western blot

Western blot was performed as previously described [34]. Briefly, tissue samples from the ipsilateral cortex of sham/TBI mouse brains were homogenized in RIPA buffer (50 mM Tris-HCl at pH 7.4,150 mM NaCl, 1% Triton X-100, 1% sodium deoxycholate, 0.1% SDS, 1 mM EDTA) with protease and phosphatase inhibitors, followed by denaturation at 95 °C for 10 min. Then the protein samples were separated by SDS-PAGE and transferred onto polyvinylidene fluoride (PVDF) membranes (Millipore). Next, the PVDF membranes were blocked with 5% bovine serum albumin for 1 h and incubated with the primary antibodies overnight, including anti-Mer antibody (1:1000, Abcam, ab184086), anti-p-Mer antibody (1:500, Abcam, ab192649), anti-STAT1 antibody (1:1000, CST, 14994), anti-p-STAT1 antibody (1: 1000, CST, 9167), anti-SOCS-1 (1:1000, Abcam, ab62584), anti-SOCS-3 (1:1000, Abcam, ab16030), and anti-GAPDH (1:5000, Abcam, ab8245). After that, the PVDF membranes were disposed of with the relevant secondary antibodies (1:5000) for 1 h at room temperature and observed using the ECL kit chemiluminescence reagents (Millipore, Billerica, MA, USA). The signals of protein bands were detected with the Chemidoc detection system and quantified using Quantity One software (Bio-Rad).

### CD11b-positive cell isolation

A magnetic-bead conjugated anti-CD11b antibody was used to isolated microglia/macrophages from injured brain tissue using magnetic-activated cell sorting (MACS) technology as previously described [18]. Ipsilateral cortical tissues from sham and CCI mice were dissociated using Neural Tissue Dissociation Kit (Miltenyi Biotec, 130-093-231) according to the manufacturer’s instructions. The dissociated brain samples were re-suspended and applied to a Strainer (70 μm). Debris in the sample was removed using Debris Removal Solution (Miltenyi Biotec, 130-109-398). Red blood cells were lysed by Red Blood Cell lysis Solution (Miltenyi Biotec, 130-094-183). Myelin was removed by Myelin Removal Beads (Miltenyi Biotec, 130-096-733). Finally, cell sample was incubated with anti-CD11b microbeads (Miltenyi Biotec, 130-049-601) and loaded onto a magnetic LS column ((Miltenyi Biotec, 130-042-401) placed in the magnetic field of a MACS Separator (Miltenyi Biotec, 130-090-976). After washing, the magnetically labeled cells were flushed out immediately by firmly pushing the plunger into the column to get CD11b positive cells, which can be used for PCR analysis.

### Immunohistochemistry and confocal microscopy analysis

Mice were anesthetized and transcardially perfused with 0.1 mmol PBS and 4% paraformaldehyde (PFA) at 3 days post-TBI. Twenty-micrometer coronal cryosections were permeabilized and incubated in 5% Donkey Serum for 1 h for blocking. Then the brain tissues were incubated in primary antibody overnight at 4 °C. The primary antibody information is as follows: anti-Mer antibody (1:400, AF591, R&D Systems), anti-CD16/32 antibody (1:200, Abcam, ab25235), anti-CD206 antibody (1:400, Abcam ab64693), anti-Iba-1 antibody (1:500, Wako, 019-19741 or 1:500, Abcam, ab5076). After the incubation overnight, the cryosections were incubated with the secondary antibodies (1:500, Jackson Immunoresearch Laboratories) for 1 h at room temperature. After that, the sections were rinsed with PBS and covered with fluorescence mounting medium with 4',6-diamidino-2-phenylindole (DAPI) (Vector Laboratories, H-1200).

For image analysis and quantification of immunofluorescent, Nissl, and Fluoro-Jade B (FJB) staining, coronal sections from bregma − 1.0 to − 3.0 mm were collected. In each animal, 5 randomly selected fields from 5 nonadjacent sections with intervals of 100 μm in the ipsilateral cortex were analyzed. The region of interest (ROI) is ~ 200 μm adjacent to the lesion boundaries in the brain lesions. For immunofluorescence analysis of ROI (450 × 350 μm^2^ per field) (as indicated in Fig. 2d, left and Suppl. Fig. 3A), the fluorescence intensity and the number of Mer^+^, CD16/32^+^, and CD206^+^ cells, as well as their colocalization with Iba-1 staining, were calculated by using ImageJ software and averaged per mouse, and each group included 6 animals [42].

### Nissl staining

Nissl staining was performed according to the manufacturer’s instructions. After 20-μm coronal sections had been deparaffinized and rehydrated, the slides were stained in Nissl Staining Solution (C0117, Beyotime) for 5 min at 37 °C. A large cell body, with abundant cytoplasm and with substantially significant levels of Nissl body, represents a normal neuron. However, some other cell forms such as a shrunken cell body, condensed nuclei, and reduced or disappearance of Nissl body represents a damaged cell. In each animal, 5 randomly selected fields (450 × 350 μm^2^ per field) in the ipsilateral cortex (Suppl. Fig. 3B) were analyzed in 5 nonadjacent sections (100 μm apart) and averaged per mouse. Cell counting was performed by using the ImageJ software, and a total of 6 mice from each group were used for quantification.

### Fluoro-Jade B (FJB) staining

The FJB staining was performed as previously described [34]. Twenty-micrometer coronal sections were first immersed in a solution containing 1% sodium hydroxide in 80% alcohol for 5 min, followed by 2 min in 70% alcohol and 2 min in distilled water. The slides were then incubated in a solution of 0.06% potassium permanganate for 10 min, rinsed in distilled water for 2 min, and incubated in a 0.0004% solution of FJB (Chemicon, Temecula, CA, USA) made in 0.1% acetic acid for 30 min. The slides were rinsed, air-dried, cleared in xylene for a minute, and coverslipped with fluorescence mounting medium with DAPI. All sections were observed and photographed under a fluorescence microscope with blue (450–490 nm) excitation light. In each animal, 5 randomly selected fields (640 × 480 μm^2^ per field) in the ipsilateral cortex (Suppl. Fig. 3C) were analyzed in 5 nonadjacent sections (100 μm apart) and averaged per mouse. Cell counting was performed by using the ImageJ software, and a total of 6 mice from each group were used for quantification.

### Statistical analysis

All data were shown as means ± standard deviation. For comparison between two groups, the *F* test was conducted to determine the similarity in the variances between the groups that are statistically compared, and statistical significance was analyzed by Student’s *t* test. For multiple comparisons, Bartlett’s test for equal variances was used to determine the variances between the multiple groups and one-way or two-way analysis of variance (ANOVA) followed by Bonferroni’s post hoc test which was used to test statistical significance. All analyses were performed using GraphPad Prism 8 software by an investigator blinded to the experimental conditions. A *p* value < 0.05 was considered as statistically significant.

## Results

### Dynamic changes in mRNA expression of microglial/macrophage M1/M2 polarization markers following TBI

**Fig. 1:**
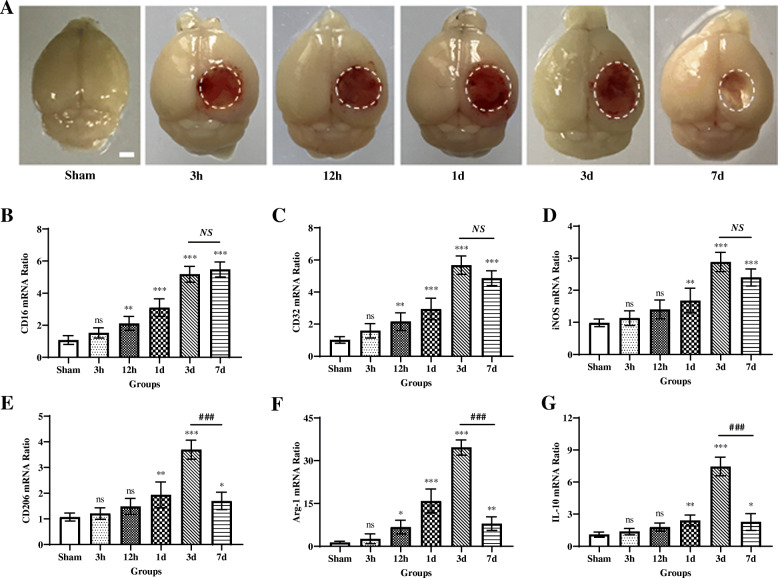
Dynamic changes in mRNA expression of microglial/macrophage M1-like and M2-like phenotypic markers following TBI. **a** Representative photographs of whole brains in the sham and different traumatic brain injury (TBI) groups (at 3 h, 12 h, 1 day, 3 days, and 7 days post-insult, respectively). Scale bar = 1 mm. Quantitative reverse transcription-polymerase chain reaction (RT-PCR) was used to assess the mRNA expression levels of microglial/macrophage M1-like and M2-like phenotypic markers in the injured cortex at 3 h, 12 h, 1 day, 3 days, and 7 days after TBI or the equivalent area of the sham-operated brains. **b**–**d** Expressions of mRNA of M1-like phenotypic markers, including CD16 (**b**), CD32 (**c**), and iNOS (**d**), were gradually increased over time from 12 h onward and remained elevated for at least 7 days after injury. **e**–**g** Expressions of mRNA of M2-like phenotypic markers, including CD206 (**e**), Arg-1 (**f**), and IL-10 (**g**), were significantly upregulated at 1 day, 12 h, and 1 day after TBI, respectively, and all peaked around day 3 post-injury, then declined dramatically on day 7, even though they did not return to baseline levels. In **b**–**g**, data are expressed as fold change. *n* = 6 mice per group. ns, nonsignificant, *p* > 0.05; *, *p* < 0.05; **, *p* < 0.01; ***, *p* < 0.001; versus sham-operated controls; NS, nonsignificant, *p* > 0.05; ###, *p* < 0.001; versus the TBI group on day 3. One-way ANOVA followed by Bonferroni’s post hoc tests

Polarized microglia/macrophages can be commonly distinguished by their expression profiles of signature surface markers and cytokines/chemokines. To evaluate the microglial/macrophage activation states following TBI (Suppl. Fig. 2A), RT-PCR was performed using total RNA extractions from the perilesional cortical tissues at 3 h, 12 h, 1 day, 3 days, and 7 days after TBI or sham operation (Fig. 1a). Levels of M1-associated markers, including CD16 (Fig. 1b), CD32 (Fig. 1c), and iNOS (Fig. 1d), were gradually increased over time from 12 h onward and remained elevated for at least 7 days after injury. The mRNA levels of tested M2-like markers, including CD206 (Fig. 1e), Arg-1 (Fig. 1f), and IL-10 (Fig. 1g), began to be significantly upregulated at 1 day, 12 h, and 1 day after TBI, respectively, and all peaked around day 3 post-injury, then declined dramatically on day 7, even though they did not return to baseline levels. These results indicated that both M1-like and M2-like polarized microglia/macrophages were activated during the acute and subacute phase response after TBI. TBI induces transient upregulation in the M2-like phenotype but causes sustained upregulation in the M1-like phenotype post-insult, which is consistent with recent reports [16, 17].

### Expression patterns and cellular localization of Mer following TBI

**Fig. 2:**
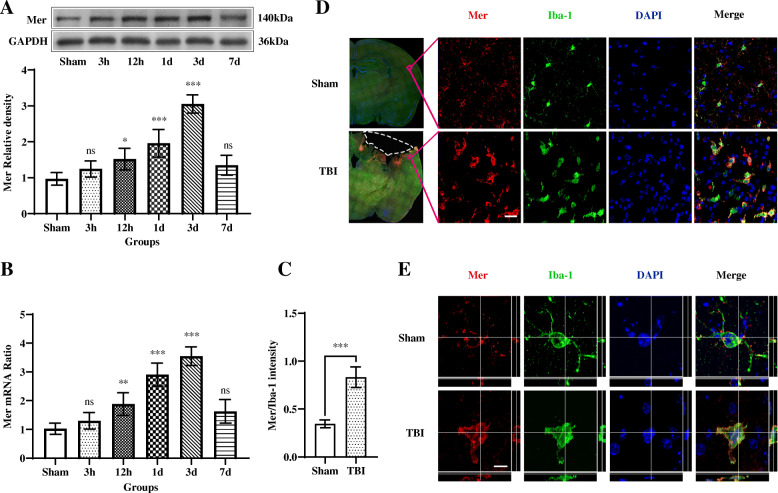
Expression patterns and cellular localization of Mer following TBI. **a** Representative immunoblots and quantification showing the expression level of Mer protein in the injured cortex at 3 h, 12 h, 1 day, 3 days, and 7 days after TBI or the equivalent area of the sham-operated brains. Data are expressed as fold change compared to sham-operated controls. *n* = 6 mice per group. **b** Quantitative RT-PCR was used to assess the mRNA expression level of Mer in the injured cortex at 3 h, 12 h, 1 day, 3 days, and 7 days after TBI or the equivalent area of the sham-operated brains. Data are expressed as fold change compared to sham-operated controls. *n* = 6 mice per group. Double immunofluorescent staining of Mer with the microglial/macrophage marker Iba-1 in the ipsilateral cerebral cortex was performed at 3 days post-TBI or sham operation. **c** Fluorescence intensity quantification of Mer expression in activated microglia/macrophages in the impacted cortical area at 3 days after TBI, compared to that in resting microglia/macrophages from sham-operated brains. *n* = 6 mice per group. **d** Low-magnification images of the brain (left) indicate the region of interest. Representative confocal images from the ipsilateral cortex (right) showing Mer was abundantly expressed in the plasma membrane of microglia/macrophages and substantially upregulated in activated microglia/macrophages following TBI. The dotted white area indicates contusion region. Scale bar = 15 μm. **e** Confocal microscopy analysis at a single-cell resolution showing Mer expression was substantially upregulated in the activated microglia/macrophages with phagocytotic morphology at 3 days post-TBI. Orthogonal views demonstrated the colocalization of Mer and Iba-1 in microglia/macrophages. Scale bar = 5 μm. In **a**, **b**, data are presented as mean ± SD; *, *p* < 0.05; **, *p* < 0.01; ***, *p* < 0.001; ns, nonsignificant, *p* > 0.05; versus sham-operated controls; one-way ANOVA followed by Bonferroni’s post hoc tests. In **c**, data are presented as mean ± SD; ***, *p* < 0.001 by Student’s *t* test

Mer plays a critical role in regulating microglial/macrophage phenotypes and properties under physiological and pathological conditions [29]. We first analyzed the levels of Mer protein and mRNA in the injured cortex at different time points after TBI (Suppl. Fig. 2A). As shown in Fig. 2a, b, both Mer protein and mRNA levels were significantly increased 12 h after TBI, peaked around day 3 and returned to the pre-injury levels around day 7. We further investigated the cellular localization and expression of Mer in microglia/macrophages in the ipsilateral cerebral cortex at 3 days after TBI, using double immunofluorescent staining of Mer and microglial/macrophage marker Iba-1. As demonstrated in Fig. 2c, e, Mer was abundantly expressed in the plasma membrane of microglia/macrophages in the sham group and substantially upregulated in the activated microglia/macrophages with phagocytotic morphology (Fig. 2d, e) near the lesion at 3 days post-TBI.

### Inhibition of Mer worsened the functional outcomes after TBI

**Fig. 3:**
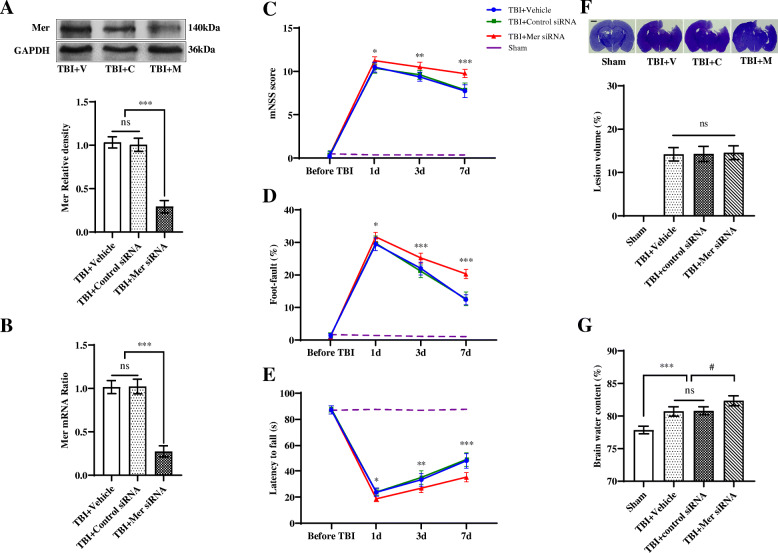
In vivo knockdown of Mer worsened the functional outcomes after TBI. **a** Western blot was used to assess the knockdown efficacy of Mer siRNA. Representative immunoblots and quantification showing Mer siRNA inhibited the expression of Mer protein in the injured cortex at 3 days post-TBI. TBI + vehicle: TBI + V, TBI + control siRNA: TBI + C, TBI + Mer siRNA: TBI + M. GAPDH: loading control. Data are expressed as fold change compared to the TBI + V group; *n* = 6 mice per group. **b** Quantitative RT-PCR analysis also showing Mer siRNA significantly inhibited the expression of Mer mRNA in the injured cortex at 3 days post-TBI. Data are expressed as fold change compared to the TBI + V group; *n* = 6 mice per group. **c**–**e** Modified neurological severity scores (mNSS) (**c**), foot-fault test (**d**), and rotarod test (**e**) were performed before and 1, 3, and 7 days after TBI. *n* = 8 mice per group. **f** Quantification of TBI-induced lesion volume at 3 days post-insult. *n* = 6 mice per group. Scale bar = 1 mm. **g** Cerebral edema was measured by brain water content. Mer siRNA significantly elevated brain edema level at 3 days post-injury, when compared to both the vehicle and control siRNA group. *n* = 8 mice per group. In **a**–**g**, data are presented as mean ± SD; *, #, *p* < 0.05; **, *p* < 0.01; ***, *p* < 0.001; ns, nonsignificant, *p* > 0.05. In **a**, **b**, **f**, **g**, one-way ANOVA followed by Bonferroni’s post hoc tests. In **c**–**e**, two-way ANOVA followed by Bonferroni’s post hoc tests

Previous studies reported that *i.c.v.* delivery of siRNA can efficiently silence specific genes in the brain [37–39]. To determine the role of Mer in the pathophysiology of TBI, we administrated Mer siRNA into the ipsilateral ventricle at 1 day before and 10 min after TBI to inhibit its expression in the brain [43] (Suppl. Fig. 2B). Data from the Western blot and RT-PCR demonstrated the knockdown efficacy of Mer siRNA was > 70% (Fig. 3a, b). Besides, we evaluated the functional outcomes following TBI using the mNNS score and foot-fault and rotarod tests performed before and 1, 3, and 7 days after TBI. When compared to the sham group, TBI mice exhibited significantly worse in all neurobehavioral tests (Fig. 3c–e). More importantly, administration of Mer siRNA significantly aggravated the neurobehavioral deficits post-injury when compared with the group that received control siRNA at days 1, 3, and 7 after TBI, based on mNNS scores (Fig. 3c), foot-fault test (Fig. 3d), and rotarod test (Fig. 3e). Specifically, research studies reported that sex differences exist in the pathophysiology of CNS injuries and diseases [44]. To evaluate sex-dimorphic responses after TBI, neurobehavioral function assessment was performed before and after TBI. Our preliminary data showed that there is no difference in behavioral tests between male and female mice at days 1, 3, and 7 after TBI, based on the mNSS score (Suppl. Fig. 4A), foot-fault test (Suppl. Fig. 4B), and rotarod test (Suppl. Fig. 4C). Also, the expression levels of Mer in the injured cortex between male and female mice were analyzed on day 3 post-injury. As shown in Suppl. Fig. 4D-E, no difference was detected in Mer protein (Suppl. Fig. 4D) and mRNA (Suppl. Fig. 4E) expression between male and female mice on day 3 after TBI.

To assess the gross pathological changes, we compared brain contusion volume and tissue edema among sham and TBI groups at 3 days post-injury. As demonstrated, TBI caused significant loss of brain tissue (Fig. 3f) and increased brain edema in the ipsilateral hemisphere (Fig. 3g). Interestingly, although Mer siRNA did not affect the brain contusion volume on day 3 after TBI (Fig. 3f), it significantly elevated brain edema level at 3 days post-injury, when compared to both the vehicle and control siRNA group (Fig. 3g). These findings suggested that Mer presence and its upregulation after injury is beneficial for the recovery after TBI.

### Mer modulated microglial/macrophage M1/M2 polarization after TBI

**Fig. 4:**
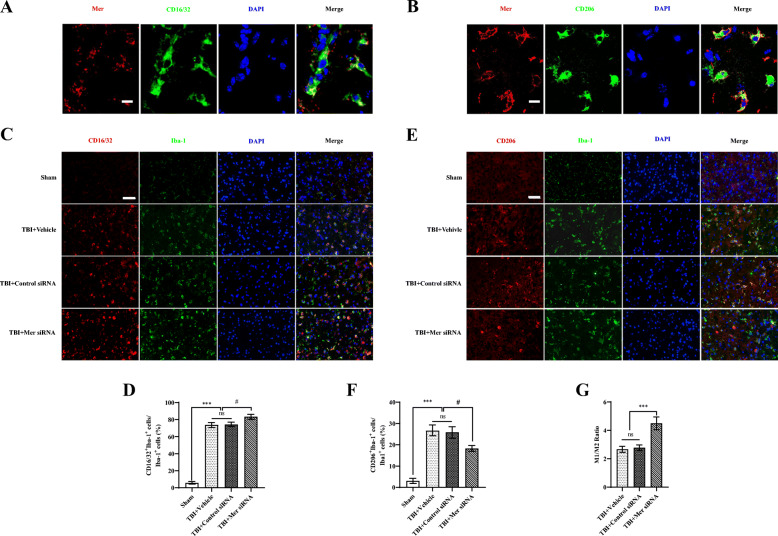
Mer modulated microglial/macrophage M1/M2 polarization after TBI. **a** Representative images of immunofluorescent staining for Mer (red), CD16/32 (green), and DAPI (blue) showing Mer was expressed in CD16/32-positive cells in the perilesional area of cortex at 3 days post-TBI. Scale bar = 10 μm. **b** Representative images of immunofluorescent staining for Mer (red), CD206 (green), and DAPI (blue) showing Mer was expressed in CD206-positive cells in the perilesional area of cortex at 3 days post-TBI. Scale bar = 10  μm. **c** Representative images of immunofluorescent staining for CD16/32 (red), Iba-1 (green), and DAPI (blue) in the ipsilateral cortex at 3 days post-TBI. Scale bar = 50 μm. **d** Quantification showing the percentage of CD16/32 and Iba-1 double-positive cells was significantly increased in the cortex on day 3 after TBI, which was further elevated in the Mer siRNA group. *n* = 6 mice per group. **e** Representative images of immunofluorescent staining for Mer (red), CD206 (green), and DAPI (blue) in the ipsilateral cortex at 3 days post-TBI. Scale bar = 50 μm. **f** Quantification showing the percentage of CD206 and Iba-1 double-positive cells increased significantly in the ipsilateral cortex on day 3 after TBI; however, it significantly decreased following Mer siRNA administration. **g** The ratio between CD16/32^+^ Iba-1^+^ M1-like cells and CD206^+^ Iba-1^+^ M2-like cells nearly doubled after Mer siRNA administration. In **d**, **f**, and **g**, data are presented as mean ± SD; #, *p* < 0.05; ***, *p* < 0.001; ns, nonsignificant, *p* > 0.05. One-way ANOVA followed by Bonferroni’s post hoc tests

To investigate the role of Mer in regulating microglial/macrophage M1/M2 polarization and neuroinflammation following TBI, we evaluated the level of Mer in both M1-like and M2-like polarized microglia/macrophages in TBI-affected cortical regions. Results from double immunofluorescent staining demonstrated that Mer was at a relatively lower level in CD16/32-positive M1-like cells (Fig. 4a and Suppl. Fig. 5A), but highly expressed in CD206-positive M2-like cell population (Fig. 4b and Suppl. Fig. 5A), in the perilesional area of cortex at 3 days post-TBI.

To specifically evaluate the effect of Mer siRNA on the M1/M2 polarization state of microglia/macrophages after TBI, CD16/32 or CD206 was co-labeled with the microglia/macrophage marker Iba-1 in the cortex surrounding the lesion cavity at 3 days post-insult, respectively. As demonstrated, the percentage of CD16/32 and Iba-1 double-positive M1-like cells was significantly increased in the cortex on day 3 after TBI, which was further elevated in the Mer siRNA group (Fig. 4c, d). On the other hand, the percentage of CD206 and Iba-1 double-positive M2-like cells was also increased significantly in the ipsilateral cortex on day 3 after TBI when compared with the sham group (Fig. 4e, f), but it was significantly decreased following Mer siRNA administration when compared with the TBI + vehicle or TBI + control siRNA group (Fig. 4e, f). In addition, the ratio between CD16/32^+^Iba-1^+^ M1-like cells and CD206^+^Iba-1^+^ M2-like cells was nearly doubled after Mer siRNA administration (Fig. 4g). Taken together, these findings indicated that Mer plays an important role in regulating microglial/macrophage M1/M2 polarization following TBI, and inhibition of Mer expression tips the balance of TBI-induced microglial/macrophage activation towards the pro-inflammatory M1-like phenotype.

### Downregulation of Mer aggravated neuronal damage and degeneration following TBI

**Fig. 5:**
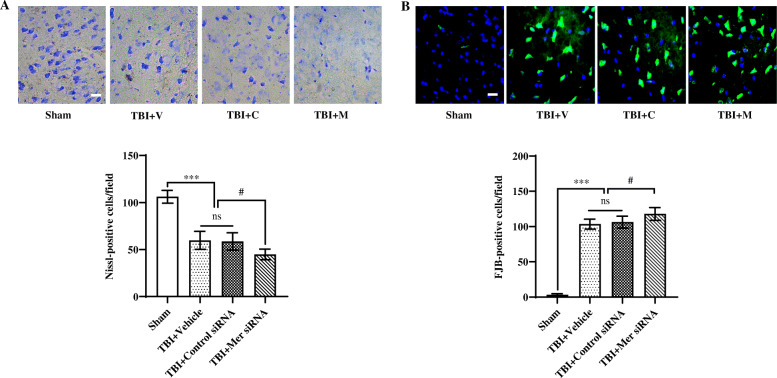
Inhibition of Mer aggravated neuronal damage and degeneration following TBI. **a** Representative images of Nissl staining in the ipsilateral cortex from the sham, TBI + vehicle (TBI + V), TBI + control siRNA (TBI + C), and TBI + Mer siRNA (TBI + M) groups, respectively. Quantification analysis showing TBI caused a significant decrease in the number of Nissl-positive cells in the ipsilateral cortex at 3 days post-TBI, and Mer siRNA application further decreased the number of Nissl-positive cells in the injured cortex after TBI. *n* = 6 mice per group. Scale bar = 20 μm. **b** Representative images of Fluoro-Jade B (FJB) staining in the ipsilateral cortex from the sham, TBI + V, TBI + C, and TBI + M groups, respectively. Quantification analysis showing TBI caused a significant increase in the number of FJB-positive cells in the ipsilateral cortex at 3 days post-TBI, and Mer siRNA application further increased the number of FJB-positive cells in the injured cortex after TBI. *n* = 6 mice per group. Scale bar = 15 μm. In **a**, **b**, data are presented as mean ± SD; #, *p* < 0.05; ***, *p* < 0.001; ns, nonsignificant, *p* > 0.05. One-way ANOVA followed by Bonferroni’s post hoc tests

To further evaluate the pathological effects of Mer knockdown following TBI, Nissl and FJB staining were employed to access the neuronal damage and degeneration following TBI. As shown, TBI caused a significant decrease in the number of Nissl-positive neurons in the perilesional cortex at 3 days post-insult (Fig. 5a). Moreover, in vivo knockdown of Mer aggravated neuronal damage following TBI, as demonstrated by the significant decrease in the number of Nissl-positive neurons in the injured cortex in the TBI + Mer siRNA group compared to the TBI + vehicle or TBI + control siRNA group (Fig. 5a). Besides, FJB staining showed that TBI induced a significant increase in the number of degenerating neurons in the injured cortex, and Mer siRNA application further increased the number of degenerating neurons in the injured cortex after TBI (Fig. 5b). These data indicated inhibition of Mer expression aggravated neuronal damage and degeneration following TBI.

### Inhibition of Mer reduced STAT1 activation and SOCS expression following TBI

**Fig. 6.**
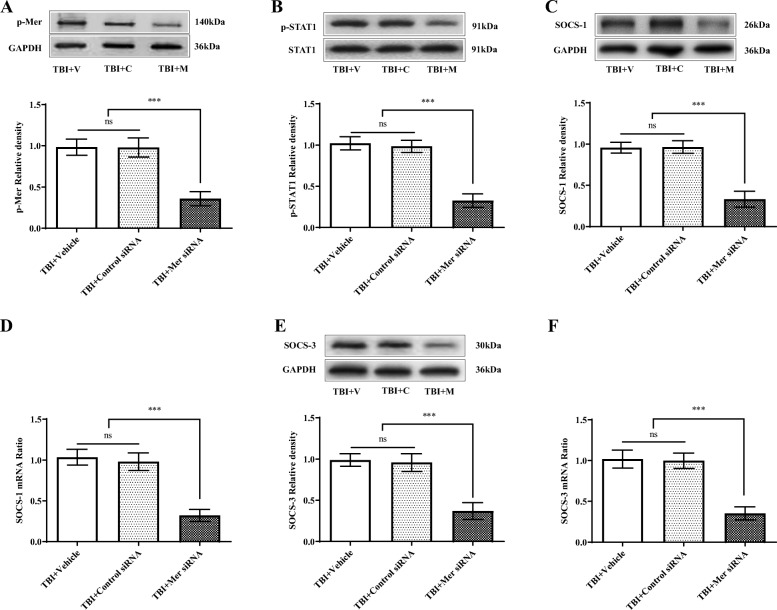
Inhibition of Mer reduced STAT1 activation and SOCS expression following TBI. **a**, **b**, **c**, **e** Representative immunoblots and quantification showing the expression of phosphorylated Mer (p-Mer) (**a**), phosphorylated STAT1 (p-STAT1) (**b**), SOCS-1 (**c**), and SOCS-3 (**e**) was significantly inhibited by Mer siRNA administration on day 3 following TBI. TBI + vehicle: TBI + V, TBI + control siRNA: TBI + C; TBI + Mer siRNA: TBI + M. GAPDH: loading control. Data are expressed as fold change compared to the TBI + V group; *n* = 6 mice per group. **d**, **f** Quantitative RT-PCR analysis showing Mer siRNA application significantly inhibited the mRNA expression of SOCS-1 (**d**) and SOCS-3 (**f**) in the injured cortex at 3 days post-TBI. Data are expressed as fold change compared to the TBI + V group; *n* = 6 mice per group. In **a**–**f**, data are presented as Mean ± SD; ns, nonsignificant, *p* > 0.05; *** *p* < 0.001. One-way ANOVA followed by Bonferroni’s post hoc tests

Previous studies indicated that Mer is a pleiotropic regulator of the innate immune system, and it regulates inflammatory responses through modulating multiple downstream cellular pathways such as STAT1/SOCS signaling [22, 25]. Activation of Mer signaling is associated with phosphorylation of the tyrosine residue 749 in the kinase domain [45]. To clarify the mechanism by which Mer interferes with microglial/macrophage M1/M2 polarization, we determined the expression of the suppressor of cytokine signaling SOCS-1 and SOCS-3 after TBI, which are key mediators of Mer signaling in regulating inflammatory responses [46, 47]. As demonstrated, the levels of phosphorylated Mer (p-Mer) (Fig. 6a) and phosphorylated STAT1 (p-STAT1) (Fig. 6b) were significantly inhibited by Mer siRNA administration on day 3 following TBI. More importantly, the protein and mRNA expression of SOCS-1 (Fig. 6c, d) and SOCS-3 (Fig. 6e, f) were significantly reduced after TBI in the Mer siRNA treatment group when compared with the vehicle or control siRNA group. These findings indicated that Mer signaling is required for activation of STAT1 and SOCS signaling pathway in the perilesional area of the cortex following TBI.

### Mer activation alleviated functional deficits following TBI

**Fig. 7:**
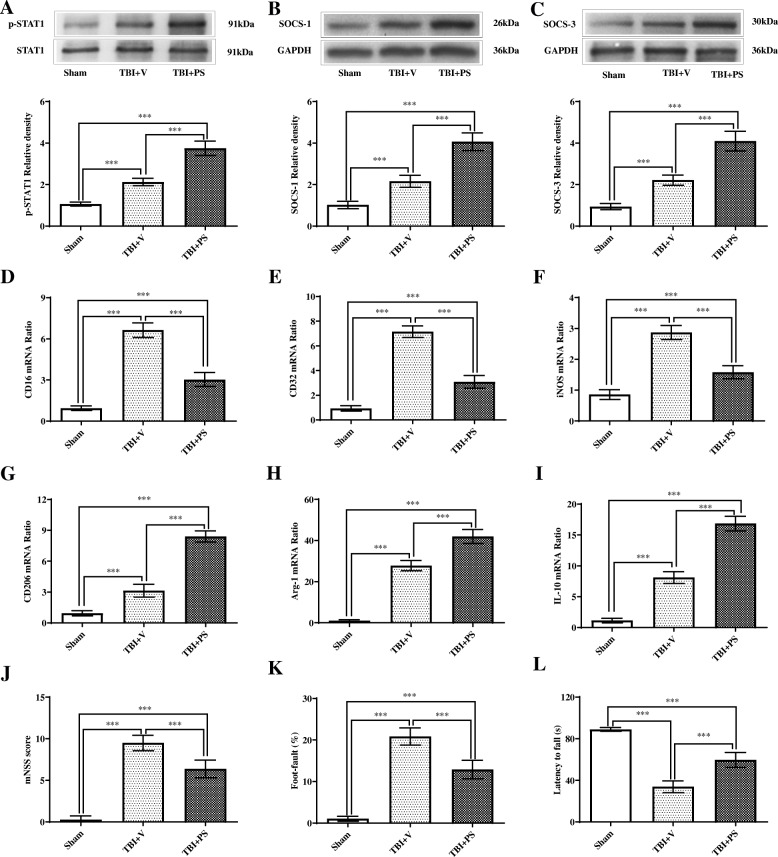
Mer activation alleviated functional deficits following TBI. **a**–**c** Representative immunoblots and quantification showing protein expression of p-STAT1 (**a**), SOCS-1 (**b**), and SOCS-3 (**c**) in the injured cortex at 3 days post-TBI; TBI + vehicle: TBI + V, TBI + recombinant protein S: TBI + PS. GAPDH: loading control. Data are expressed as fold change compared to the sham group; *n* = 6 mice per group. **d**–**i** Quantitative RT-PCR analysis of MACS-sorted CD11b-positive cells showing mRNA expression of CD16 (**d**), CD32 (**e**), iNOS (**f**), CD206 (**g**), Arg-1 (**h**), and IL-10 (**i**) in the injured cortex at 3 days post-TBI. GAPDH: loading control. Data are expressed as fold change compared to the sham group; *n* = 6 mice per group. **j**–**l** Modified neurological severity scores (mNSS) (**j**), foot-fault test (**k**), and rotarod test (**l**) were performed at 3 days after TBI. *n* = 8 mice per group. In **a**–**l**, data are presented as mean ± SD; ***, *p* < 0.001. One-way ANOVA followed by Bonferroni’s post hoc tests

**Fig. 8:**
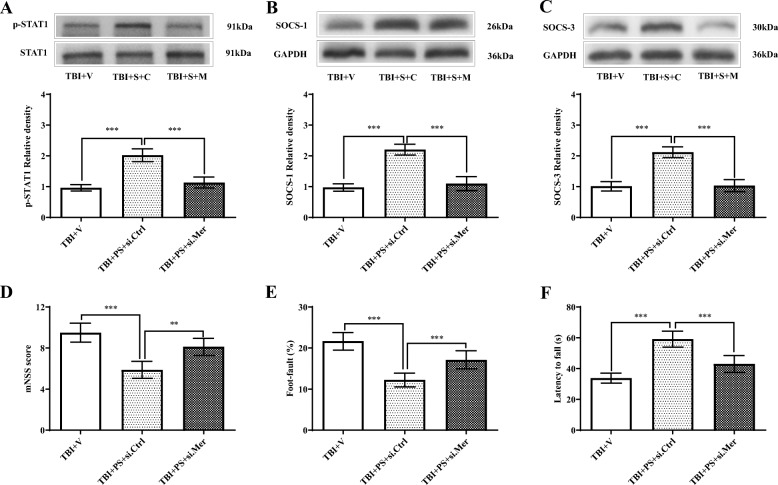
Inhibition of Mer abolished PS-mediated functional improvements following TBI. **a**–**c** Representative immunoblots and quantification showing protein expression of p-STAT1 (**a**), SOCS-1 (**b**), and SOCS-3 (**c**) in the injured cortex at 3 days after TBI; TBI + vehicle: TBI + V, TBI + PS + control siRNA (si.Ctrl): TBI + S + C, TBI + S + Mer siRNA (si.Mer): TBI + S + M. GAPDH: loading control. Data are expressed as fold change compared to the TBI + V group; *n* = 6 mice per group. **d**–**f** Modified neurological severity scores (mNSS) (**d**), foot-fault test (**e**), and rotarod test (**f**) were performed at 3 days after TBI. *n* = 8 mice per group. In **a**–**f**, data are presented as mean ± SD; ***, *p* < 0.001. One-way ANOVA followed by Bonferroni’s post hoc tests

To further determine the role of Mer following TBI, administration of recombinant PS (a ligand and activator of Mer) was performed at 1 h, 1 day, and 2 days after TBI, respectively (Suppl. Fig. 2C and 2D) [48]. Western blot assay revealed PS treatment caused a significant increase of p-STAT1 (Fig. 7a), SOCS-1 (Fig. 7b), and SOCS-3 (Fig. 7c) expression when compared with the vehicle group. PCR assay revealed that expression of M1-associated markers, including CD16 (Suppl. Fig. 5B), CD32 (Suppl. Fig. 5C), and iNOS (Suppl. Fig. 5D), was heavily suppressed by PS treatment when compared with the vehicle group. By contrast, the tested M2-like markers, including CD206 (Suppl. Fig. 5E), Arg-1 (Suppl. Fig. 5F), and IL-10 (Suppl. Fig. 5G), were further upregulated after PS administration. Consistently, PCR assay of MACS-sorted CD11b-positive cells revealed that expression levels of M1-associated markers, including CD16 (Fig. 7d), CD32 (Fig. 7e), and iNOS (Fig. 7f), were largely suppressed by PS treatment when compared with vehicle treatment. By contrast, the tested M2-associated markers, including CD206 (Fig. 7g), Arg-1 (Fig. 7h), and IL-10 (Fig. 7i), were further increased after PS administration. Furthermore, TBI-induced brain edema was reduced by PS treatment at 3 days post-injury (Suppl. Fig. 6A). Also, TBI-induced neuronal damage was attenuated by PS treatment, as demonstrated by the significant increase in the number of Nissl-positive neurons (Suppl. Fig. 6B-C) and the significant decrease in the number of FJB-positive degenerating neurons (Suppl. Fig. 6D-E) in the injured cortex in the TBI + PS group compared to the TBI + vehicle group. Moreover, PS treatment significantly alleviated neurobehavioral deficits on day 3 post-injury, based on the mNNS score (Fig. 7j), foot-fault test (Fig. 7k), and rotarod test (Fig. 7l). However, Mer siRNA administration abolished PS-induced changes in p-STAT1 level (Fig. 8a) and expression levels of SOCS-1 (Fig. 8b) and SOCS-3 (Fig. 8c) in TBI mice. In addition, the mNNS score (Fig. 8d), foot-fault test (Fig. 8e), and rotarod test (Fig. 8f) showed worsened neurological outcomes in mice from the PS + Mer siRNA group when compared with the PS + control siRNA treatment group. Taken together, these findings suggested that Mer promoted STAT1-mediated upregulation of SOCS expression, alleviated secondary brain injury, and improved functional outcomes following TBI.

## Discussion

**Fig. 9:**
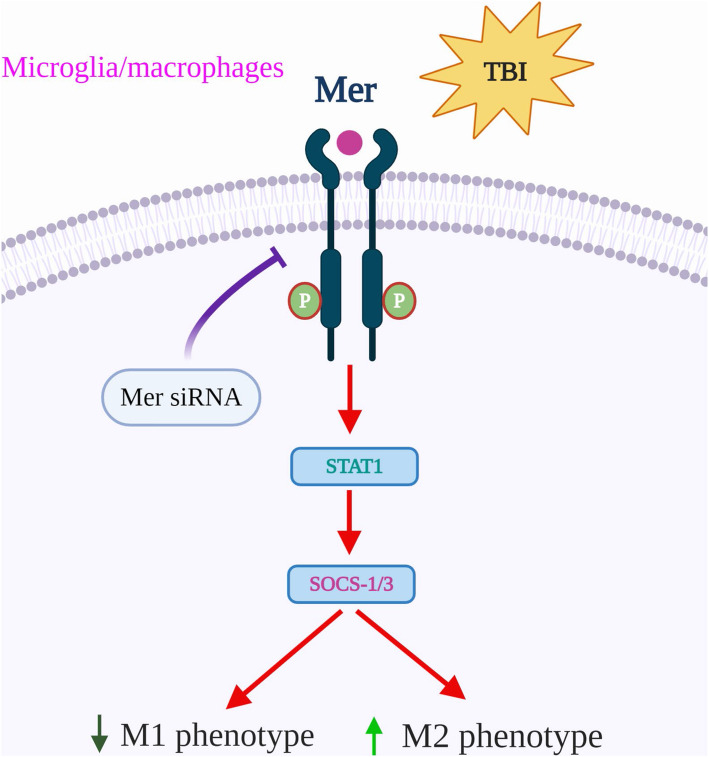
The proposed mechanism of Mer in regulating microglial/macrophage M1/M2 polarization and neuroinflammation following TBI. In the setting of TBI, Mer is upregulated and exerts protective effects via modulating microglial/macrophage M1/M2 polarization and neuroinflammation after injury. Specifically, activation of Mer signaling facilitates STAT1-mediated SOCS expression, which increases microglial/macrophage M2-like polarization while decreases M1-like polarization following TBI. In contrast, inhibition of Mer by siRNA markedly suppresses STAT1/SOCS signaling, thus decreasing microglial/macrophage M2-like polarization while increasing M1-like polarization after TBI

Neuroinflammation is conceived as an important manipulable aspect of secondary brain injury following TBI [13]. Here, we demonstrate that Mer upregulation provides neuroprotective effects in a CCI mouse model of TBI via regulating microglial/macrophage M1/M2 polarization and neuroinflammation (Fig. 9). Mechanistically, Mer mediates activation of the STAT1/SOCS signaling pathway. Inhibition of Mer markedly decreases microglial/macrophage M2-like polarization while it increases M1-like polarization, thus aggravating secondary brain damage and functional deficits after TBI.

TBI is an evolving neurological event, and it heavily contributes to both acute injuries and chronic neurodegenerative cascades post-insult [49, 50]. This disease is challenging to be treated because of its heterogeneous nature and complex pathogenic cascades [51, 52]. Of note, microglial/macrophage activation and neuroinflammation are hallmark features of TBI pathophysiology [8]. As the resident innate immune cells in the CNS, microglia usually respond within minutes toward the sites of damage, where they can even sustain for many years following TBI [13]. Initially, these responses allow them to scavenge debris, promote tissue remodeling and repair, and protect the brain from secondary injury after TBI [53]. However, abnormal activation of microglia can interfere with endogenous repair mechanisms and drives inflammatory damage after TBI [53]. In addition, peripherally derived macrophages actively participate in acute neuroinflammatory responses after TBI [12]. Indeed, microglia/macrophages have multi-dimensional activation states in CNS diseases depending on different pathophysiological conditions [54]. They can become polarized ranging from the classic M1-like phenotype to an alternative M2-like phenotype after CNS injuries including TBI [8, 55]. The M1-like response is presumed to be pro-inflammatory [15], whereas the M2-like phenotype owns anti-inflammatory effects [56]. Multiple molecular pathways, such as STAT, nuclear factor-κB (NF-κB), and interferon regulatory factor (IRF), are involved in the regulation of M1/M2 phenotypic transitions [57–59]. Preclinical evidence indicated that mixed phenotypes are present in the pathological processes of TBI, which offer opportunities for therapeutic interventions [60]. In the current study, we also found that TBI induces transient upregulation in the M2-like phenotype but causes sustained upregulation in the M1-like phenotype post-insult, which is consistent with other findings [16, 17]. Modulating microglial/macrophage M1/M2 polarization via regulating those intrinsic molecular switches will facilitate the repair activity of microglia/macrophages and promote neurovascular network restoration after TBI. Notably, microglia/macrophages are endowed with spectacular plasticity, allowing them to acquire multiple phenotypes and thereby fulfill numerous activities in health and disease [61]. The existing M1/M2 paradigm, based on expression analysis of a small subset of genes (labeled M1 or M2), is inadequate for accurate description of the true diversity of microglia/macrophages during complex disease environments such as TBI [13]. Indeed, existing evidence suggests that microglia/macrophages do not strictly polarize to an “M1-only” or “M2-only” phenotype in response to the multifaceted inflammatory milieu following TBI [20]. In contrast, these cells are dually labeled with “M1/M2” markers and display a mixed phenotype in both the microenvironment and within the same cell after injury [12, 21]. Thus, a binary M1/M2 polarization paradigm is not adequate to define the inflammatory profiles following TBI [21]. And it is not feasible to classify the cells (e.g., M1, M2a, M2b, M2c) depending on few selectively chosen inflammatory markers [21, 56], while exploring the role and underlying regulatory mechanisms of these markers in the context of TBI-induced neuroinflammation would be more favorable [20, 62]. Also, multiple methodological approaches that can reveal transcriptomic and proteomic profiling of microglia/macrophages are indeed needed in future studies, which will help tremendously in our understanding of microglial/macrophage diversity and development of better-targeted therapies for a variety of neurological disease conditions including TBI [13, 61].

Mer, an important member of the TAM family, is predominantly expressed on myeloid-derived hematopoietic cells and functions as a pleiotropic inhibitor of the innate immune response [63]. RNA sequencing data demonstrated that Mer mRNA is highly enriched in microglia in the CNS [64]. In contrast, the mRNA expression levels of the other two family members, namely Axl and Tyro3, are very low in microglia [64]. Moreover, PS and Gas6, two best-characterized ligands, can bind the Ig1 and Ig2 domains of Mer via their C-terminal regions [65]. Upon ligand stimulation, Mer can be activated and initiate classic ligand-inducible dimerization, causing receptor autophosphorylation, recruitment of signaling proteins with SH2 or PTB domains, and activation of downstream pathways. As an example, activation of Mer can induce SOCS expression and inhibit inflammation through regulating type I interferon receptor (IFNAR)-associated STAT1 pathway [22]. Genetic targeting of Mer can lead to persistent inflammation and tissue damage in a mouse model of acetaminophen-induced acute liver failure [66]. Also, Mer can mediate recognition and subsequent efferocytosis of apoptotic cells to prevent immune responses [67]. In an in vivo murine model of allergic airway inflammation, Mer-mediated apoptotic eosinophil clearance by phagocytes contributes to resolution of allergic airway inflammation [68]. In a murine model of experimental myocardial infarction, deficiency of Mer in a subset of myocardial monocytes suppresses the removal of dying cardiac cells, delays inflammation resolution, and reduces systolic performance after myocardial infarction [69]. Proteolytic cleavage of macrophage Mer reduces efferocytosis and inhibits plaque resolution in atherosclerosis [70]. In contrast, in fat-fed low-density lipoprotein receptor-deficient mice whose myeloid cells expressing a cleavage-resistant variant of Mer, higher expression of Mer in macrophage contributes to improved efferocytosis, smaller necrotic cores, thicker fibrous caps, and increased ratios of pro-resolving versus pro-inflammatory lipid mediators in the atherosclerotic lesions [70]. These findings indicate Mer plays a prominent role in the resolution of inflammation through multiple approaches. Furthermore, previous studies have demonstrated that TAM receptors play complex roles in modulating neuronal migration and neurogenesis, synaptic plasticity, microglial activation and efferocytosis, and peripheral nerve repair [71, 72], resulting in potential interests in neuroinflammatory and neurodegenerative diseases such as Alzheimer’s disease [73], Parkinson’s disease [29], and multiple sclerosis [74]. Our study added new knowledge about the role of this pathway in TBI and shows that Mer is upregulated acutely in the injured cortex after injury, which exerts an effect on microglial/macrophage polarization towards an M2-like phenotype after TBI. And proper stimulation of Mer signaling by its recombinant ligands such as PS can regulate microglial/macrophage activities and neuroinflammatory responses after TBI, which contributes to alleviation of secondary brain damage and sensorimotor impairments after injury. On the other hand, due to the complex and multiscale nature of secondary injury cascades following TBI, therapies designed to target multiple mechanisms of this disease will likely be needed [75]. Thus, molecular targets like Mer, which can regulate multiple cellular processes, may be targeted for therapy in TBI and deserve further investigation.

Noteworthily, some limitations in this study should not be ignored. First, the available molecular markers are not specific enough to sufficiently distinguish microglia from other closely related myeloid subsets, particularly monocyte-derived macrophages [64]. It is true that portions of peripheral macrophages can infiltrate into the injured brain after TBI [76, 77]. Thus, the impact of Mer-mediated regulation of inflammatory responses in microglia and infiltrating macrophages following TBI was not distinctively evaluated during the result interpretation, and it is possible that infiltrating macrophages could be driving some of the effects. In fact, the crucial roles of peripheral infiltrated myeloid cells in TBI neuropathology have been investigated [12, 78–80]. As an example, Morganti et al. reported that TBI induces a robust response involving the recruitment and accumulation of peripheral CCR2^+^ macrophages into the injured brain parenchyma, which significantly contributes to neuroinflammatory sequelae and cognitive dysfunction after injury [12]. By contrast, targeting the subset of CCR2^+^ macrophages with a selective antagonist CCX872 heavily reduces the accumulation of these cells, decreases their inflammatory and neurotoxic profiles, and ameliorates hippocampal-related cognitive dysfunction after TBI [12]. Interestingly, it demonstrated that age can exaggerate the recruitment of peripheral CCR2^+^ macrophages to the injured parenchyma after TBI, which exerts a non-redundant and contributing role to TBI-induced neuroinflammation in the aged brain [78, 79]. Moreover, it reported that peripherally derived myeloid cells (CD45^hi^CD11b^+^) play an important role in initiating acute neuroinflammatory responses in the injured brain after TBI, which are sexually dimorphic and exaggerated in males compared to female mice [81]. Noteworthily, the relative contributions of resident microglia and infiltrating peripheral myeloid cells to TBI pathology were not adequately discriminated in a number of experimental studies, which should be largely examined in future research [81]. Also, we used whole cortical samples but not isolated microglia/macrophages from sham and CCI mice for mRNA analysis of dynamic changes, and those tested factors can partially be produced by other cells such as neurons and astrocytes within the contused cortex. Thus, in order to demonstrate the selective upregulation of M1/M2-like markers in microglia/macrophages, isolation of these cells from the sham-injured and TBI brain for flow cytometry and image analyses will be favorable in future studies.

Additionally, several studies reported that sex difference underlies variations of microglial/macrophage activation and sexually dimorphic neuroinflammatory responses following TBI [82, 83], while other studies indicated that sex manipulation does not affect inflammatory responses and brain injury after TBI [84]. Of note, a vast majority of preclinical studies have been conducted just using male animals to look at the inflammatory responses to TBI, while increasing evidence indicates that sex differences may have important implications for TBI outcome and potential therapeutic development [85]. Contrary to our expectations, we failed to detect any differences in behavioral tests between male and female mice after TBI in the preliminary study, based on the mNSS score, foot-fault test, and rotarod test. The small animal numbers used in the current study as well as potentially inadequate sensitivity of these behavioral tests may underlie the negative results, which still needs in-depth investigation in further research. Besides, we did not find any difference in the levels of Mer protein and mRNA expression in the perilesional cortex between male and female mice on day 3 after TBI. Also, Hilliard et al. examined the effect of male-female differences on the expression of Mer in the human blood samples and found that no significant difference in Mer expression was reached between female and male subjects [86]. To date, information regarding sexual dimorphism in Mer expression is still scarce and has yet to be explored. But it should not ignore the sex difference between males and females, which would be an important biological variable in CNS injury and disease [83, 87]. And sexual dimorphism in sex hormone levels (e.g., estrogen, progesterone, and testosterone), microglial/macrophage properties, and neuroinflammatory responses may contribute to the differences between the two sexes after TBI [44, 88]. Thus, more studies are required to decipher how sex differences affect microglial/macrophage activities, neuroinflammation, and neurofunctional outcomes after TBI. And more data are certainly warranted before any definite conclusion about the relationship among sex, sex hormones, and microglia/macrophages after TBI can be drawn [44].

## Conclusions

In conclusion, our study demonstrated that Mer is upregulated and exerts beneficial effects in the acute stage of TBI via modulating microglial/macrophage M1/M2 polarization. Inhibition of Mer markedly decreases microglial/macrophage M2-like polarization while increases M1-like polarization, which contributes to the aggravation of secondary brain damage and exaggeration of sensorimotor deficits after TBI.

## Supplementary Information

**Additional file 1: Supplementary Figure 1.** **(A-B)** The controlled cortical impact (CCI) model of TBI and experimental parameters in mice. **(C)** A schematic map showing the location of TBI in mice. The center of impact was located at 2 mm medial-lateral (ML) and -2 mm anterior-posterior (AP) to bregma. **(D-E)** The intracerebroventricular (i.c.v) injection and experimental parameters in mice. **(F)** A schematic map showing coordinates of *i.c.v.* injection in mice. The stereotaxic coordinates are 1 mm ML and -0.25 mm AP to bregma, and 2.5 mm dorsoventral (DV) below the skull. LV: lateral ventricle.

**Additional file 2: Supplementary Figure 2.** Experimental design and animal groups. **(A)** Western blot (WB) and Real-Time Polymerase Chain Reaction (RT-PCR) were performed to evaluate the expression profiles of Mer and M1/M2 polarization markers at different time points after TBI (including 3 h, 12 h, 1 d, 3 d, and 7 d), as well as in the sham group; Besides, immunofluorescence was performed on day 3 after injury. **(B)** An *in vivo* knockdown of Mer siRNA was adopted to evaluate the role of Mer in regulating microglial/macrophage M1/M2 polarization after TBI. Mice were randomly distributed into sham, TBI + Vehicle, TBI + Control siRNA, and TBI + Mer siRNA groups. Intracerebroventricular injection (*i.c.v.*) of siRNA was performed 1 d before and 10 min after TBI. The neurobehavioral functions were assessed before as well as 1, 3, 7 d after TBI. The peri-injured cerebral cortex from each group and the equivalent area in the sham-operated mice was collected for RT-PCR, WB, and immunohistochemistry analysis at 3 d after TBI. Also, contusion volume, brain edema, neuronal damage and degeneration were measured at 3 d after injury. **(C)** To evaluate the effect of PS on regulating STAT1/SOCSs pathway after TBI, mice were randomly distributed into Sham, TBI + Vehicle, and TBI + recombinant protein S (PS) groups. PS (0.2 mg/kg) was administered via the tail vein at 1 h, 1 d, and 2 d after the CCI. WB, RT-PCR, MACS, neuronal damage and degeneration, brain edema, and neurobehavioral assessments were conducted on day 3 post-injury. **(D)** Mice were randomly distributed into TBI + Vehicle, TBI + PS + Control siRNA, and TBI + PS + Mer siRNA groups. Mer siRNA (*i.c.v.*) was administrated 1 d before and 10 min after TBI, and PS was administered via the tail vein at 1 h, 1 d, and 2 d after the injury. WB and neurobehavioral assessments were conducted on day 3 post-injury.

**Additional file 3: Supplementary Figure 3.** **(A)** Low-magnification images indicate the region of interest for immunofluorescent staining of CD16/32 or CD206 (red), Iba-1 (Green) and DAPI (blue) in the ipsilateral cortex from the sham, TBI + Vehicle, TBI + Control siRNA and TBI+Mer siRNA groups on day 3 post-injury, respectively. **(B)** Low-magnification images indicate the region of interest for Nissl staining in the ipsilateral cortex from the sham, TBI + Vehicle, TBI + Control siRNA and TBI+Mer siRNA groups on day 3 post-injury, respectively. **(C)** Low-magnification images indicate the region of interest for Fluoro-Jade B (FJB) staining in the ipsilateral cortex from the sham, TBI + Vehicle, TBI + Control siRNA and TBI+Mer siRNA groups on day 3 post-injury, respectively. In **A-C**, the red dotted area indicates contusion region. * indicates region of interest. Scale bar = 1 mm.

**Additional file 4: Supplementary Figure 4.** **(A)** Modified neurological severity scores (mNSS), **(B)** foot-fault test, and **(C)** rotarod test were performed before and 1, 3, and 7 d after TBI. n = 8 mice per group. **(D)** Representative immunoblots and quantification showing the expression level of Mer protein in the injured cortex at 3 d after TBI or the equivalent area of the sham-operated brains. Data are expressed as fold change compared to sham-operated controls. n = 6 mice per group. M: male; F: female. **(E)** Quantitative RT-PCR was used to assess the mRNA expression level of Mer in the injured cortex at 3 d after TBI or the equivalent area of the sham-operated brains. Data are expressed as fold change compared to sham-operated controls. n = 6 mice per group. In **A-E**, data are presented as mean ± SD; ***, *p* < 0.001; ns, non-significant, *p* > 0.05. two-way ANOVA followed by Bonferroni’s post-hoc tests.

**Additional file 5: Supplementary Figure 5.** **(A)** Fluorescence intensity quantification of Mer expression in CD16/32- and CD206- positive cells in the impacted ipsilateral cortical area at 3 d after TBI. n = 6 per group. **(B-G)** Quantitative RT-PCR analysis showing mRNA expression of CD16 **(B)**, CD32 **(C)**, iNOS **(D)**, CD206 **(E)**, Arg-1 **(F)**, IL-10 **(G)** in the injured cortex at 3 d post-TBI. GAPDH: loading control. Data are expressed as fold change compared to the sham group; n = 6 mice per group. In **A**, data are presented as Mean ± SD; ***, *p* < 0.001 by Student’s t-test. In **B-G**, data are presented as Mean ± SD; ***, *p* < 0.001. one-way ANOVA followed by Bonferroni’s post-hoc tests.

**Additional file 6: Supplementary Figure 6.** **(A)** Cerebral edema was measured by brain water content. Quantification analysis showing TBI increased brain edema in the ipsilateral hemisphere on day 3 after TBI, which is significantly alleviated after PS treatment. n = 8 mice per group. **(B)** Representative images of Nissl staining in the ipsilateral cortex from the sham, TBI + Vehicle (TBI + V), TBI + protein S (TBI + PS) groups, respectively. **(C)** Quantification analysis showing TBI caused a significant decrease in the number of Nissl-positive cells in the ipsilateral cortex at 3 d post-TBI, and PS application significantly increased the number of Nissl-positive cells in the injured cortex after TBI. n = 6 mice per group. Scale bar = 20 μm. **(D)** Representative images of Fluoro-Jade B (FJB) staining in the ipsilateral cortex from the sham, TBI + V, TBI + PS groups, respectively. **(E)** Quantification analysis showing TBI caused a significant increase in the number of FJB-positive cells in the ipsilateral cortex at 3 d post-TBI, and PS application significantly decreased the number of FJB-positive cells in the injured cortex after TBI. n = 6 mice per group. Scale bar = 15 μm. In **A**, **C**, **E**, data are presented as Mean ± SD; **, *p* < 0.01; ***, *p* < 0.001. one-way ANOVA followed by Bonferroni’s post-hoc tests.

## Data Availability

The authors confirm that all data underlying the findings are fully available without restriction. All relevant data are within the paper and its supporting information files.

## Abbreviations

ANOVA: Analysis of variance
Arg-1: Arginase 1
CCI: Controlled cortical impact
CNS: Central nervous system diseases
DAPI: 4’,6-Diamidino-2-phenylindole
FJB: Fluoro-Jade B
Gas6: Growth arrest-specific factor 6
IFNAR: Type I interferon receptor
IL-1β: Interleukin-1β
IL-10: Interleukin-10
iNOS: Inducible nitric oxide synthase
IRF: Interferon regulatory factor
MACS: Magnetic-activated cell sorting
Mer: Myeloid-epithelial-reproductive tyrosine kinase
mNSS: Modified neurological severity scores
NF-κB: Nuclear factor-κB
PVDF: Polyvinylidene fluoride
PS: Protein S
RT-PCR: Reverse transcription-polymerase chain reaction
SOCS: Suppressor of cytokine signaling
STAT1: Signal transducer and activator of transcription 1
TAM: Tyro-Axl-Mer
TBI: Traumatic brain injury
TGF-β: Transforming growth factor-β
TNF-α: Tumor necrosis factor-α

## References

[1] GBD 2016 Neurology Collaborators (2019). Global, regional, and national burden of neurological disorders, 1990-2016: a systematic analysis for the Global Burden of Disease Study 2016. Lancet Neurol.

[2] Capizzi A, Woo J, Verduzco-Gutierrez M (2020). Traumatic brain injury: an overview of epidemiology, pathophysiology, and medical management. Med Clin North Am.

[3] Dewan MC, Rattani A, Gupta S, Baticulon RE, Hung Y-C, Punchak M (2019). Estimating the global incidence of traumatic brain injury. J Neurosurg..

[4] Blennow K, Hardy J, Zetterberg H (2012). The neuropathology and neurobiology of traumatic brain injury. Neuron..

[5] Wilson L, Stewart W, Dams-O’Connor K, Diaz-Arrastia R, Horton L, Menon DK (2017). The chronic and evolving neurological consequences of traumatic brain injury. Lancet Neurol..

[6] Stocchetti N, Zanier ER (2016). Chronic impact of traumatic brain injury on outcome and quality of life: a narrative review. Crit Care Lond Engl..

[7] Loane DJ, Faden AI (2010). Neuroprotection for traumatic brain injury: translational challenges and emerging therapeutic strategies. Trends Pharmacol Sci..

[8] Simon DW, McGeachy MJ, Bayır H, Clark RSB, Loane DJ, Kochanek PM (2017). The far-reaching scope of neuroinflammation after traumatic brain injury. Nat Rev Neurol..

[9] Johnson VE, Stewart JE, Begbie FD, Trojanowski JQ, Smith DH, Stewart W (2013). Inflammation and white matter degeneration persist for years after a single traumatic brain injury. Brain J Neurol..

[10] Smith C, Gentleman SM, Leclercq PD, Murray LS, Griffin WST, Graham DI (2013). The neuroinflammatory response in humans after traumatic brain injury. Neuropathol Appl Neurobiol..

[11] Witcher KG, Eiferman DS, Godbout JP (2015). Priming the inflammatory pump of the CNS after traumatic brain injury. Trends Neurosci..

[12] Morganti JM, Jopson TD, Liu S, Riparip L-K, Guandique CK, Gupta N (2015). CCR2 antagonism alters brain macrophage polarization and ameliorates cognitive dysfunction induced by traumatic brain injury. J Neurosci Off J Soc Neurosci..

[13] Jassam YN, Izzy S, Whalen M, McGavern DB, El Khoury J (2017). Neuroimmunology of traumatic brain injury: time for a paradigm shift. Neuron..

[14] Pisanu A, Lecca D, Mulas G, Wardas J, Simbula G, Spiga S (2014). Dynamic changes in pro- and anti-inflammatory cytokines in microglia after PPAR-γ agonist neuroprotective treatment in the MPTPp mouse model of progressive Parkinson’s disease. Neurobiol Dis..

[15] Loane DJ, Kumar A (2016). Microglia in the TBI brain: The good, the bad, and the dysregulated. Exp Neurol..

[16] Kumar A, Alvarez-Croda D-M, Stoica BA, Faden AI, Loane DJ (2016). Microglial/macrophage polarization dynamics following traumatic brain injury. J Neurotrauma..

[17] Wang G, Zhang J, Hu X, Zhang L, Mao L, Jiang X (2013). Microglia/macrophage polarization dynamics in white matter after traumatic brain injury. J Cereb Blood Flow Metab Off J Int Soc Cereb Blood Flow Metab..

[18] Kumar A, Barrett JP, Alvarez-Croda D-M, Stoica BA, Faden AI, Loane DJ (2016). NOX2 drives M1-like microglial/macrophage activation and neurodegeneration following experimental traumatic brain injury. Brain Behav Immun..

[19] Wang G, Shi Y, Jiang X, Leak RK, Hu X, Wu Y (2015). HDAC inhibition prevents white matter injury by modulating microglia/macrophage polarization through the GSK3β/PTEN/Akt axis. Proc Natl Acad Sci U S A..

[20] Rosi S (2016). A polarizing view on posttraumatic brain injury inflammatory response. Brain Circ..

[21] Morganti JM, Riparip L-K, Rosi S (2016). Call off the dog(ma): M1/M2 polarization is concurrent following traumatic brain injury. PloS One..

[22] Rothlin CV, Carrera-Silva EA, Bosurgi L, Ghosh S (2015). TAM receptor signaling in immune homeostasis. Annu Rev Immunol..

[23] Cai B, Kasikara C, Doran AC, Ramakrishnan R, Birge RB, Tabas I (2018). MerTK signaling in macrophages promotes the synthesis of inflammation resolution mediators by suppressing CaMKII activity. Sci Signal..

[24] Choi J-Y, Seo JY, Yoon Y-S, Lee Y-J, Kim H-S, Kang JL (2015). Mer signaling increases the abundance of the transcription factor LXR to promote the resolution of acute sterile inflammation. Sci Signal..

[25] Lee Y-J, Han J-Y, Byun J, Park H-J, Park E-M, Chong YH (2012). Inhibiting Mer receptor tyrosine kinase suppresses STAT1, SOCS1/3, and NF-κB activation and enhances inflammatory responses in lipopolysaccharide-induced acute lung injury. J Leukoc Biol..

[26] Cai B, Thorp EB, Doran AC, Subramanian M, Sansbury BE, Lin C-S (2016). MerTK cleavage limits proresolving mediator biosynthesis and exacerbates tissue inflammation. Proc Natl Acad Sci U S A..

[27] Ji R, Tian S, Lu HJ, Lu Q, Zheng Y, Wang X (2013). TAM receptors affect adult brain neurogenesis by negative regulation of microglial cell activation. J Immunol Baltim Md 1950.

[28] Healy LM, Perron G, Won S-Y, Michell-Robinson MA, Rezk A, Ludwin SK (2016). MerTK Is a functional regulator of myelin phagocytosis by human myeloid cells. J Immunol Baltim Md 1950.

[29] Fourgeaud L, Través PG, Tufail Y, Leal-Bailey H, Lew ED, Burrola PG (2016). TAM receptors regulate multiple features of microglial physiology. Nature..

[30] Myers KV, Amend SR, Pienta KJ (2019). Targeting Tyro3, Axl and MerTK (TAM receptors): implications for macrophages in the tumor microenvironment. Mol Cancer..

[31] Ubil E, Caskey L, Holtzhausen A, Hunter D, Story C, Earp HS (2018). Tumor-secreted Pros1 inhibits macrophage M1 polarization to reduce antitumor immune response. J Clin Invest..

[32] Kim S-Y, Lim E-J, Yoon Y-S, Ahn Y-H, Park E-M, Kim H-S (2016). Liver X receptor and STAT1 cooperate downstream of Gas6/Mer to induce anti-inflammatory arginase 2 expression in macrophages. Sci Rep..

[33] Zizzo G, Hilliard BA, Monestier M, Cohen PL (2012). Efficient clearance of early apoptotic cells by human macrophages requires M2c polarization and MerTK induction. J Immunol Baltim Md 1950..

[34] Wu H, Shao A, Zhao M, Chen S, Yu J, Zhou J (2016). Melatonin attenuates neuronal apoptosis through up-regulation of K(+) -Cl(-) cotransporter KCC2 expression following traumatic brain injury in rats. J Pineal Res..

[35] Zhu D, Wang Y, Singh I, Bell RD, Deane R, Zhong Z (2010). Protein S controls hypoxic/ischemic blood-brain barrier disruption through the TAM receptor Tyro3 and sphingosine 1-phosphate receptor. Blood..

[36] Liu D, Guo H, Griffin JH, Fernández JA, Zlokovic BV (2003). Protein S confers neuronal protection during ischemic/hypoxic injury in mice. Circulation..

[37] Nikolakopoulou AM, Montagne A, Kisler K, Dai Z, Wang Y, Huuskonen MT (2019). Pericyte loss leads to circulatory failure and pleiotrophin depletion causing neuron loss. Nat Neurosci..

[38] Godinho BMDC, Henninger N, Bouley J, Alterman JF, Haraszti RA, Gilbert JW (2018). Transvascular delivery of hydrophobically modified siRNAs: gene silencing in the rat brain upon disruption of the blood-brain barrier. Mol Ther J Am Soc Gene Ther..

[39] Ma Q, Chen S, Hu Q, Feng H, Zhang JH, Tang J (2014). NLRP3 inflammasome contributes to inflammation after intracerebral hemorrhage. Ann Neurol..

[40] Zheng J, Sun Z, Liang F, Xu W, Lu J, Shi L (2019). AdipoRon attenuates neuroinflammation after intracerebral hemorrhage through AdipoR1-AMPK pathway. Neuroscience..

[41] Batsaikhan B, Wang J-Y, Scerba MT, Tweedie D, Greig NH, Miller JP (2019). Post-injury neuroprotective effects of the thalidomide analog 3,6’-dithiothalidomide on traumatic brain injury. Int J Mol Sci..

[42] Zhu H-L, Liu Z-P, Yang W-Y, Dong D-W, Zhao Y, Yang B (2018). Liraglutide ameliorates β-amyloid deposits and secondary damage in the ipsilateral thalamus and sensory deficits after focal cerebral infarction in rats. Front Neurosci..

[43] Tong L-S, Shao A-W, Ou Y-B, Guo Z-N, Manaenko A, Dixon BJ (2017). Recombinant Gas6 augments Axl and facilitates immune restoration in an intracerebral hemorrhage mouse model. J Cereb Blood Flow Metab Off J Int Soc Cereb Blood Flow Metab..

[44] Caplan HW, Cox CS, Bedi SS (2017). Do microglia play a role in sex differences in TBI?. J Neurosci Res..

[45] Ling L, Templeton D, Kung HJ (1996). Identification of the major autophosphorylation sites of Nyk/Mer, an NCAM-related receptor tyrosine kinase. J Biol Chem..

[46] van den Brand BT, Abdollahi-Roodsaz S, Vermeij EA, Bennink MB, Arntz OJ, Rothlin CV (2013). Therapeutic efficacy of Tyro3, Axl, and Mer tyrosine kinase agonists in collagen-induced arthritis. Arthritis Rheum..

[47] Zhen Y, Finkelman FD, Shao W-H (2018). Mechanism of Mer receptor tyrosine kinase inhibition of glomerular endothelial cell inflammation. J Leukoc Biol..

[48] Liao D, Wang X, Li M, Lin PH, Yao Q, Chen C (2009). Human protein S inhibits the uptake of AcLDL and expression of SR-A through Mer receptor tyrosine kinase in human macrophages. Blood..

[49] Sivanandam TM, Thakur MK (2012). Traumatic brain injury: a risk factor for Alzheimer’s disease. Neurosci Biobehav Rev..

[50] Smith DH, Johnson VE, Trojanowski JQ, Stewart W (2019). Chronic traumatic encephalopathy - confusion and controversies. Nat Rev Neurol..

[51] Cruz-Haces M, Tang J, Acosta G, Fernandez J, Shi R (2017). Pathological correlations between traumatic brain injury and chronic neurodegenerative diseases. Transl Neurodegener..

[52] Puntambekar SS, Saber M, Lamb BT, Kokiko-Cochran ON (2018). Cellular players that shape evolving pathology and neurodegeneration following traumatic brain injury. Brain Behav Immun..

[53] Morganti-Kossmann MC, Semple BD, Hellewell SC, Bye N, Ziebell JM (2019). The complexity of neuroinflammation consequent to traumatic brain injury: from research evidence to potential treatments. Acta Neuropathol (Berl)..

[54] Li Q, Barres BA (2018). Microglia and macrophages in brain homeostasis and disease. Nat Rev Immunol..

[55] Hu X, Leak RK, Shi Y, Suenaga J, Gao Y, Zheng P (2015). Microglial and macrophage polarization—new prospects for brain repair. Nat Rev Neurol..

[56] Cherry JD, Olschowka JA, O’Banion MK (2014). Neuroinflammation and M2 microglia: the good, the bad, and the inflamed. J Neuroinflammation..

[57] Kobayashi K, Imagama S, Ohgomori T, Hirano K, Uchimura K, Sakamoto K (2013). Minocycline selectively inhibits M1 polarization of microglia. Cell Death Dis..

[58] Qin H, Yeh W-I, De Sarno P, Holdbrooks AT, Liu Y, Muldowney MT (2012). Signal transducer and activator of transcription-3/suppressor of cytokine signaling-3 (STAT3/SOCS3) axis in myeloid cells regulates neuroinflammation. Proc Natl Acad Sci U S A..

[59] Tanaka T, Murakami K, Bando Y, Yoshida S (2015). Interferon regulatory factor 7 participates in the M1-like microglial polarization switch. Glia..

[60] Xu H, Wang Z, Li J, Wu H, Peng Y, Fan L (2017). The polarization states of microglia in TBI: a new paradigm for pharmacological intervention. Neural Plast..

[61] Stratoulias V, Venero JL, Tremblay M-È, Joseph B (2019). Microglial subtypes: diversity within the microglial community. EMBO J..

[62] Hsieh CL, Kim CC, Ryba BE, Niemi EC, Bando JK, Locksley RM (2013). Traumatic brain injury induces macrophage subsets in the brain. Eur J Immunol..

[63] Zhang B, Lu H, Jiang A, Wu H, Fang L, Lv Y (2019). MerTK downregulates lipopolysaccharide-induced inflammation through SOCS1 protein but does not affect phagocytosis of Escherichia coli in macrophages. Inflammation..

[64] Bennett ML, Bennett FC, Liddelow SA, Ajami B, Zamanian JL, Fernhoff NB (2016). New tools for studying microglia in the mouse and human CNS. Proc Natl Acad Sci U S A..

[65] Lemke G (2013). Biology of the TAM receptors. Cold Spring Harb Perspect Biol..

[66] Triantafyllou E, Pop OT, Possamai LA, Wilhelm A, Liaskou E, Singanayagam A (2018). MerTK expressing hepatic macrophages promote the resolution of inflammation in acute liver failure. Gut..

[67] DeBerge M, Yeap XY, Dehn S, Zhang S, Grigoryeva L, Misener S (2017). MerTK Cleavage on resident cardiac macrophages compromises repair after myocardial ischemia reperfusion injury. Circ Res..

[68] Felton JM, Lucas CD, Dorward DA, Duffin R, Kipari T, Vermeren S (2018). Mer-mediated eosinophil efferocytosis regulates resolution of allergic airway inflammation. J Allergy Clin Immunol..

[69] Wan E, Yeap XY, Dehn S, Terry R, Novak M, Zhang S (2013). Enhanced efferocytosis of apoptotic cardiomyocytes through myeloid-epithelial-reproductive tyrosine kinase links acute inflammation resolution to cardiac repair after infarction. Circ Res..

[70] Cai B, Thorp EB, Doran AC, Sansbury BE, Daemen MJAP, Dorweiler B (2017). MerTK receptor cleavage promotes plaque necrosis and defective resolution in atherosclerosis. J Clin Invest..

[71] Tondo G, Perani D, Comi C (2019). TAM receptor pathways at the crossroads of neuroinflammation and neurodegeneration. Dis Markers..

[72] Shafit-Zagardo B, Gruber RC, DuBois JC (2018). The role of TAM family receptors and ligands in the nervous system: from development to pathobiology. Pharmacol Ther..

[73] Savage JC, Jay T, Goduni E, Quigley C, Mariani MM, Malm T (2015). Nuclear receptors license phagocytosis by trem2+ myeloid cells in mouse models of Alzheimer’s disease. J Neurosci Off J Soc Neurosci..

[74] Bellan M, Pirisi M, Sainaghi PP (2016). The Gas6/TAM system and multiple sclerosis. Int J Mol Sci..

[75] Somayaji MR, Przekwas AJ, Gupta RK (2018). Combination therapy for multi-target manipulation of secondary brain injury mechanisms. Curr Neuropharmacol..

[76] Katsumoto A, Miranda AS, Butovsky O, Teixeira AL, Ransohoff RM, Lamb BT (2018). Laquinimod attenuates inflammation by modulating macrophage functions in traumatic brain injury mouse model. J Neuroinflammation..

[77] Takada S, Sakakima H, Matsuyama T, Otsuka S, Nakanishi K, Norimatsu K (2020). Disruption of Midkine gene reduces traumatic brain injury through the modulation of neuroinflammation. J Neuroinflammation..

[78] Morganti JM, Riparip L-K, Chou A, Liu S, Gupta N, Rosi S (2016). Age exacerbates the CCR2/5-mediated neuroinflammatory response to traumatic brain injury. J Neuroinflammation..

[79] Chou A, Krukowski K, Morganti JM, Riparip L-K, Rosi S (2018). Persistent infiltration and impaired response of peripherally-derived monocytes after traumatic brain injury in the aged brain. Int J Mol Sci..

[80] Lee S, Mattingly A, Lin A, Sacramento J, Mannent L, Castel M-N (2016). A novel antagonist of p75NTR reduces peripheral expansion and CNS trafficking of pro-inflammatory monocytes and spares function after traumatic brain injury. J Neuroinflammation..

[81] Doran SJ, Ritzel RM, Glaser EP, Henry RJ, Faden AI, Loane DJ (2019). Sex differences in acute neuroinflammation after experimental traumatic brain injury are mediated by infiltrating myeloid Cells. J Neurotrauma..

[82] Mangold CA, Wronowski B, Du M, Masser DR, Hadad N, Bixler GV (2017). Sexually divergent induction of microglial-associated neuroinflammation with hippocampal aging. J Neuroinflammation..

[83] Villapol S, Loane DJ, Burns MP (2017). Sexual dimorphism in the inflammatory response to traumatic brain injury. Glia..

[84] Bruce-Keller AJ, Dimayuga FO, Reed JL, Wang C, Angers R, Wilson ME (2007). Gender and estrogen manipulation do not affect traumatic brain injury in mice. J Neurotrauma..

[85] Späni CB, Braun DJ, Van Eldik LJ (2018). Sex-related responses after traumatic brain injury: Considerations for preclinical modeling. Front Neuroendocrinol..

[86] Hilliard BA, Zizzo G, Ulas M, Linan MK, Schreiter J, Cohen PL (2014). Increased expression of Mer tyrosine kinase in circulating dendritic cells and monocytes of lupus patients: correlations with plasma interferon activity and steroid therapy. Arthritis Res Ther..

[87] Gölz C, Kirchhoff FP, Westerhorstmann J, Schmidt M, Hirnet T, Rune GM (2019). Sex hormones modulate pathogenic processes in experimental traumatic brain injury. J Neurochem..

[88] Kerr N, Dietrich DW, Bramlett HM, Raval AP (2019). Sexually dimorphic microglia and ischemic stroke. CNS Neurosci Ther..

