# Supplementary material for: Mer regulates microglial/macrophage M1/M2 polarization and alleviates neuroinflammation following traumatic brain injury
Source: J Neuroinflammation. 2021 Jan 5;18:2. doi: 10.1186/s12974-020-02041-7 (PMC7787000; doi:10.1186/s12974-020-02041-7)
Supplement: Supplementary file 1 — Additional file 1: Supplementary Figure 1. (A-B) The controlled cortical impact (CCI) model of TBI and experimental parameters in mice. (C) A schematic map showing the location of TBI in mice. The center of impact was located at 2 mm medial-lateral (ML) and -2 mm anterior-posterior (AP) to bregma. (D-E) The intracerebroventricular (i.c.v) injection and experimental parameters in mice. (F) A schematic map showing coordinates of i.c.v. injection in mice. The stereotaxic coordinates are 1 mm ML and -0.25 mm AP to bregma, and 2.5 mm dorsoventral (DV) below the skull. LV: lateral ventricle. [file 12974_2020_2041_MOESM1_ESM.pdf]

Supplementary data

A

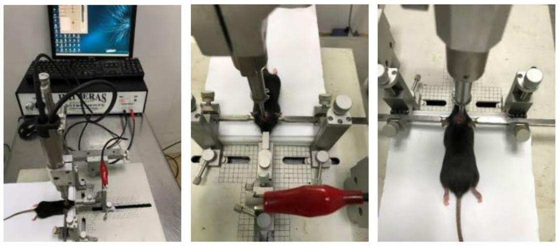

B

Experimental parameters	
Impact tip diameter	3mm
Impact velocity	3m/s
Impact duration	0.15s
Impact depth	2 mm

C

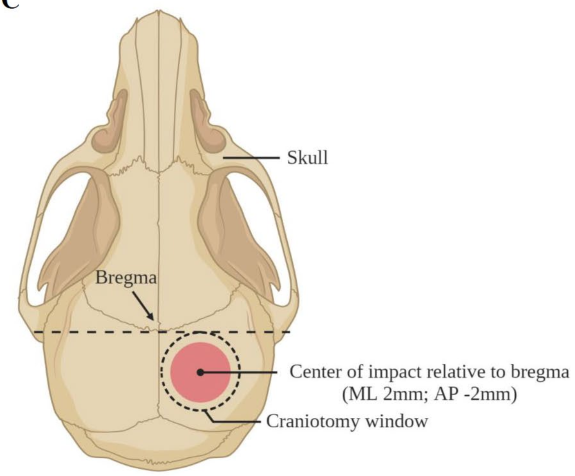

D

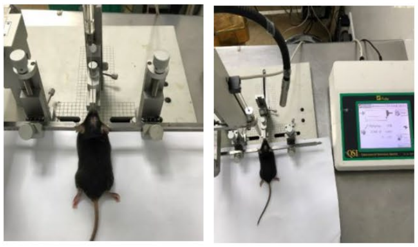

E

Experimental parameters	
Stereotactic location	ML 1mm; AP -0.25 mm; DV 2.5mm
Injection volume	2 μl
Injection velocity	0.5 μl/min

F

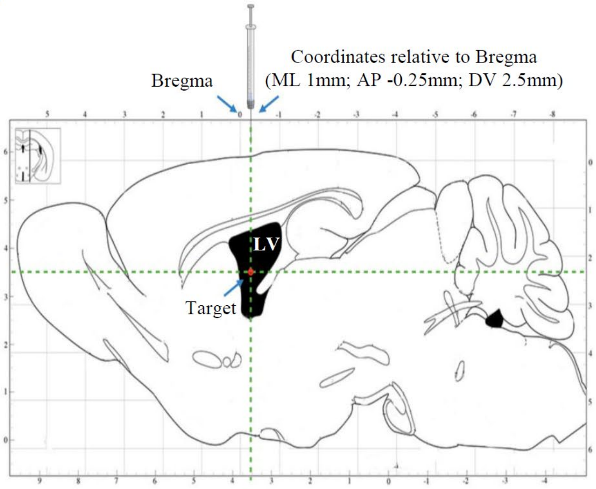

Supplementary Figure 1. (A-B) The controlled cortical impact (CCI) model of TBI and experimental parameters in mice. **(C)** A schematic map showing the location of TBI in mice. The center of impact was located at 2 mm medial-lateral (ML) and -2 mm anterior-posterior (AP) to bregma. **(D-E)** The intracerebroventricular (i.c.v) injection and experimental parameters in mice. **(F)** A schematic map showing coordinates of *i.c.v.* injection in mice. The stereotaxic coordinates are 1 mm ML and -0.25 mm AP to bregma, and 2.5 mm dorsoventral (DV) below the skull. LV: lateral ventricle.
