# Supplementary material for: Mer regulates microglial/macrophage M1/M2 polarization and alleviates neuroinflammation following traumatic brain injury
Source: J Neuroinflammation. 2021 Jan 5;18:2. doi: 10.1186/s12974-020-02041-7 (PMC7787000; doi:10.1186/s12974-020-02041-7)
Supplement: Supplementary file 2 — Additional file 2: Supplementary Figure 2. Experimental design and animal groups. (A) Western blot (WB) and Real-Time Polymerase Chain Reaction (RT-PCR) were performed to evaluate the expression profiles of Mer and M1/M2 polarization markers at different time points after TBI (including 3 h, 12 h, 1 d, 3 d, and 7 d), as well as in the sham group; Besides, immunofluorescence was performed on day 3 after injury. (B) An in vivo knockdown of Mer siRNA was adopted to evaluate the role of Mer in regulating microglial/macrophage M1/M2 polarization after TBI. Mice were randomly distributed into sham, TBI + Vehicle, TBI + Control siRNA, and TBI + Mer siRNA groups. Intracerebroventricular injection (i.c.v.) of siRNA was performed 1 d before and 10 min after TBI. The neurobehavioral functions were assessed before as well as 1, 3, 7 d after TBI. The peri-injured cerebral cortex from each group and the equivalent area in the sham-operated mice was collected for RT-PCR, WB, and immunohistochemistry analysis at 3 d after TBI. Also, contusion volume, brain edema, neuronal damage and degeneration were measured at 3 d after injury. (C) To evaluate the effect of PS on regulating STAT1/SOCSs pathway after TBI, mice were randomly distributed into Sham, TBI + Vehicle, and TBI + recombinant protein S (PS) groups. PS (0.2 mg/kg) was administered via the tail vein at 1 h, 1 d, and 2 d after the CCI. WB, RT-PCR, MACS, neuronal damage and degeneration, brain edema, and neurobehavioral assessments were conducted on day 3 post-injury. (D) Mice were randomly distributed into TBI + Vehicle, TBI + PS + Control siRNA, and TBI + PS + Mer siRNA groups. Mer siRNA (i.c.v.) was administrated 1 d before and 10 min after TBI, and PS was administered via the tail vein at 1 h, 1 d, and 2 d after the injury. WB and neurobehavioral assessments were conducted on day 3 post-injury. [file 12974_2020_2041_MOESM2_ESM.pdf]

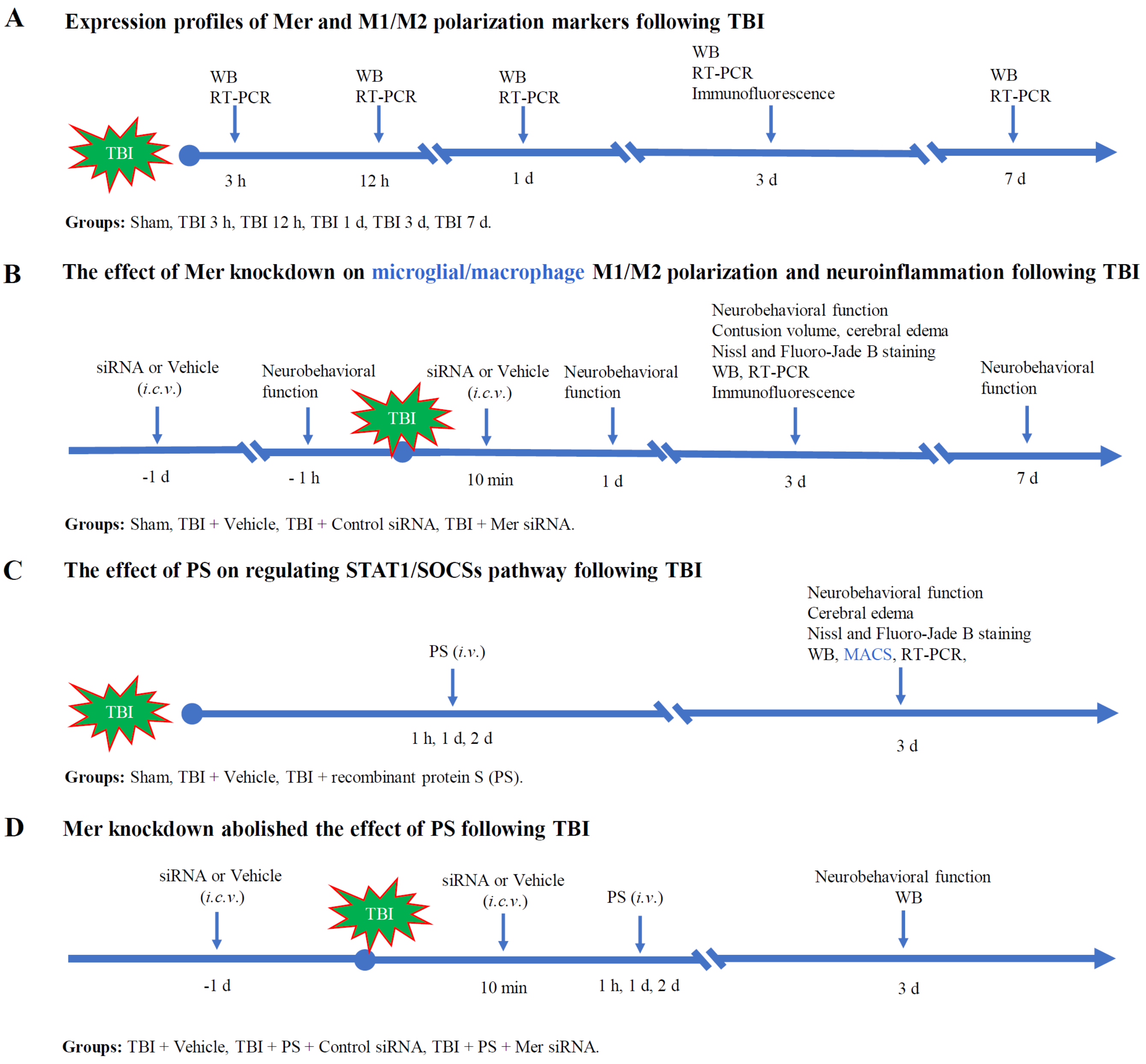

Supplementary Figure 2. Experimental design and animal groups. **(A)** Western blot (WB) and Real-Time Polymerase Chain Reaction (RT-PCR) were performed to evaluate the expression profiles of Mer and M1/M2 polarization markers at different time points after TBI (including 3 h, 12 h, 1 d, 3 d, and 7 d)**,** as well as in the sham group; Besides, immunofluorescence was performed on day 3 after injury. **(B)** An *in vivo* knockdown of Mer siRNA was adopted to evaluate the role of Mer in regulating microglial/macrophage M1/M2 polarization after TBI. Mice were randomly distributed into sham, TBI + Vehicle, TBI + Control siRNA, and TBI + Mer siRNA groups. Intracerebroventricular injection (*i.c.v.*) of siRNA was performed 1 d before and 10 min after TBI. The neurobehavioral functions were assessed before as well as 1, 3, 7 d after TBI. The peri-injured cerebral cortex from each group and the equivalent area in the sham-operated mice was collected for RT-PCR, WB, and immunohistochemistry analysis at 3 d after TBI. Also, contusion volume, brain edema, neuronal damage and degeneration were measured at 3 d after injury. **(C)** To evaluate the effect of PS on regulating STAT1/SOCSs

pathway after TBI, mice were randomly distributed into Sham, TBI + Vehicle, and TBI + recombinant protein S (PS) groups. PS (0.2 mg/kg) was administered via the tail vein at 1 h, 1 d, and 2 d after the CCI. WB, RT-PCR, MACS, neuronal damage and degeneration, brain edema, and neurobehavioral assessments were conducted on day 3 post-injury. **(D)** Mice were randomly distributed into TBI + Vehicle, TBI + PS + Control siRNA, and TBI + PS + Mer siRNA groups. Mer siRNA (*i.c.v.*) was administrated 1 d before and 10 min after TBI, and PS was administered via the tail vein at 1 h, 1 d, and 2 d after the injury. WB and neurobehavioral assessments were conducted on day 3 post-injury.
