# Supplementary material for: Mer regulates microglial/macrophage M1/M2 polarization and alleviates neuroinflammation following traumatic brain injury
Source: J Neuroinflammation. 2021 Jan 5;18:2. doi: 10.1186/s12974-020-02041-7 (PMC7787000; doi:10.1186/s12974-020-02041-7)
Supplement: Supplementary file 3 — Additional file 3: Supplementary Figure 3. (A) Low-magnification images indicate the region of interest for immunofluorescent staining of CD16/32 or CD206 (red), Iba-1 (Green) and DAPI (blue) in the ipsilateral cortex from the sham, TBI + Vehicle, TBI + Control siRNA and TBI+Mer siRNA groups on day 3 post-injury, respectively. (B) Low-magnification images indicate the region of interest for Nissl staining in the ipsilateral cortex from the sham, TBI + Vehicle, TBI + Control siRNA and TBI+Mer siRNA groups on day 3 post-injury, respectively. (C) Low-magnification images indicate the region of interest for Fluoro-Jade B (FJB) staining in the ipsilateral cortex from the sham, TBI + Vehicle, TBI + Control siRNA and TBI+Mer siRNA groups on day 3 post-injury, respectively. In A-C, the red dotted area indicates contusion region. * indicates region of interest. Scale bar = 1 mm. [file 12974_2020_2041_MOESM3_ESM.pdf]

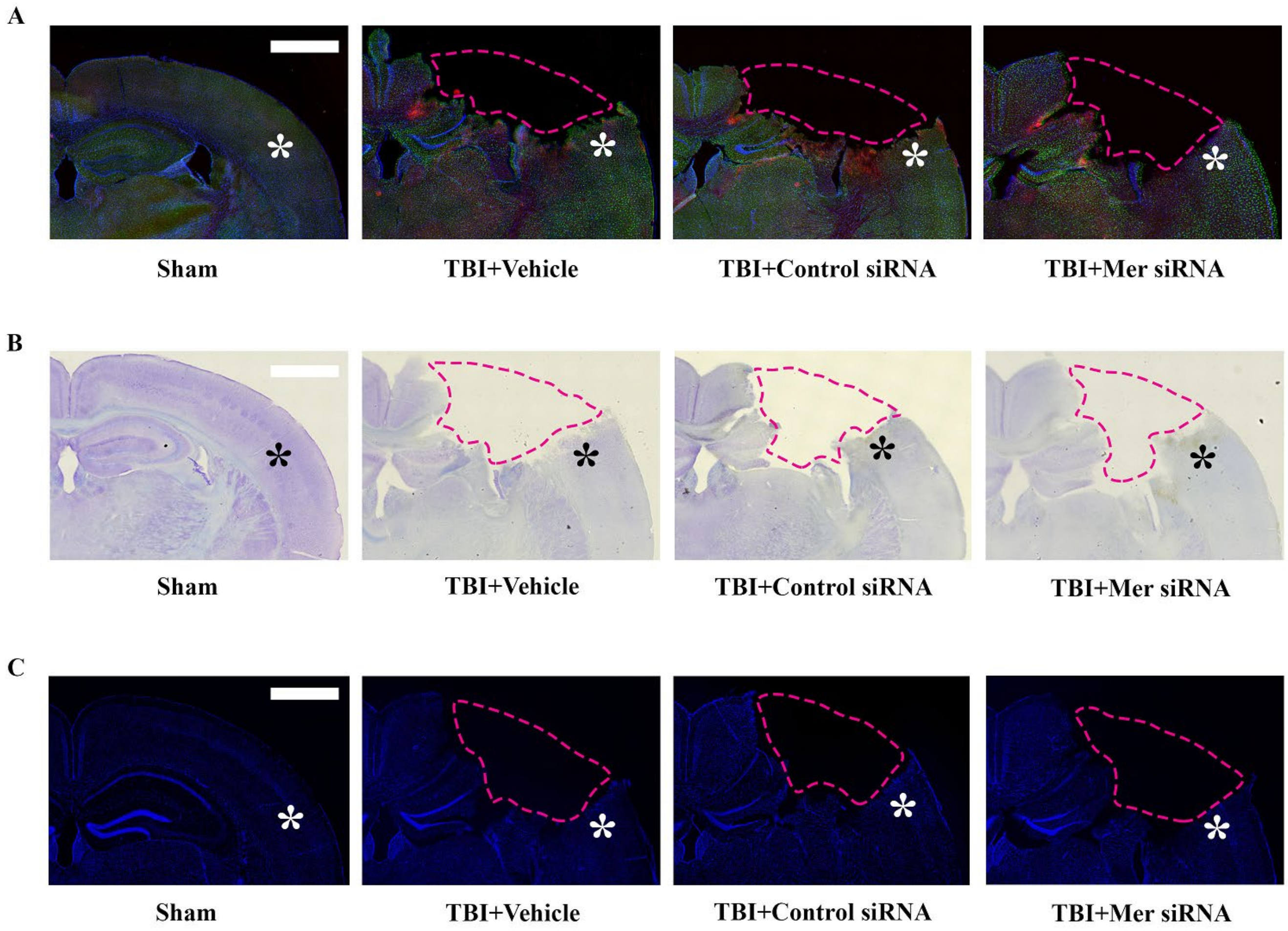

Supplementary Figure 3. **(A)** Low-magnification images indicate the region of interest for immunofluorescent staining of CD16/32 or CD206 (red), Iba-1 (Green) and DAPI (blue) in the ipsilateral cortex from the sham, TBI + Vehicle, TBI + Control siRNA and TBI+Mer siRNA groups on day 3 post-injury, respectively. **(B)** Low-magnification images indicate the region of interest for Nissl staining in the ipsilateral cortex from the sham, TBI + Vehicle, TBI + Control siRNA and TBI+Mer siRNA groups on day 3 post-injury, respectively. **(C)** Low-magnification images indicate the region of interest for Fluoro-Jade B (FJB) staining in the ipsilateral cortex from the sham, TBI + Vehicle, TBI + Control siRNA and TBI+Mer siRNA groups on day 3 post-injury, respectively. In **A-C**, the red dotted area indicates contusion region. * indicates region of interest. Scale bar = 1 mm.
