# Supplementary material for: Mer regulates microglial/macrophage M1/M2 polarization and alleviates neuroinflammation following traumatic brain injury
Source: J Neuroinflammation. 2021 Jan 5;18:2. doi: 10.1186/s12974-020-02041-7 (PMC7787000; doi:10.1186/s12974-020-02041-7)
Supplement: Supplementary file 4 — Additional file 4: Supplementary Figure 4. (A) Modified neurological severity scores (mNSS), (B) foot-fault test, and (C) rotarod test were performed before and 1, 3, and 7 d after TBI. n = 8 mice per group. (D) Representative immunoblots and quantification showing the expression level of Mer protein in the injured cortex at 3 d after TBI or the equivalent area of the sham-operated brains. Data are expressed as fold change compared to sham-operated controls. n = 6 mice per group. M: male; F: female. (E) Quantitative RT-PCR was used to assess the mRNA expression level of Mer in the injured cortex at 3 d after TBI or the equivalent area of the sham-operated brains. Data are expressed as fold change compared to sham-operated controls. n = 6 mice per group. In A-E, data are presented as mean ± SD; ***, p < 0.001; ns, non-significant, p > 0.05. two-way ANOVA followed by Bonferroni’s post-hoc tests. [file 12974_2020_2041_MOESM4_ESM.pdf]

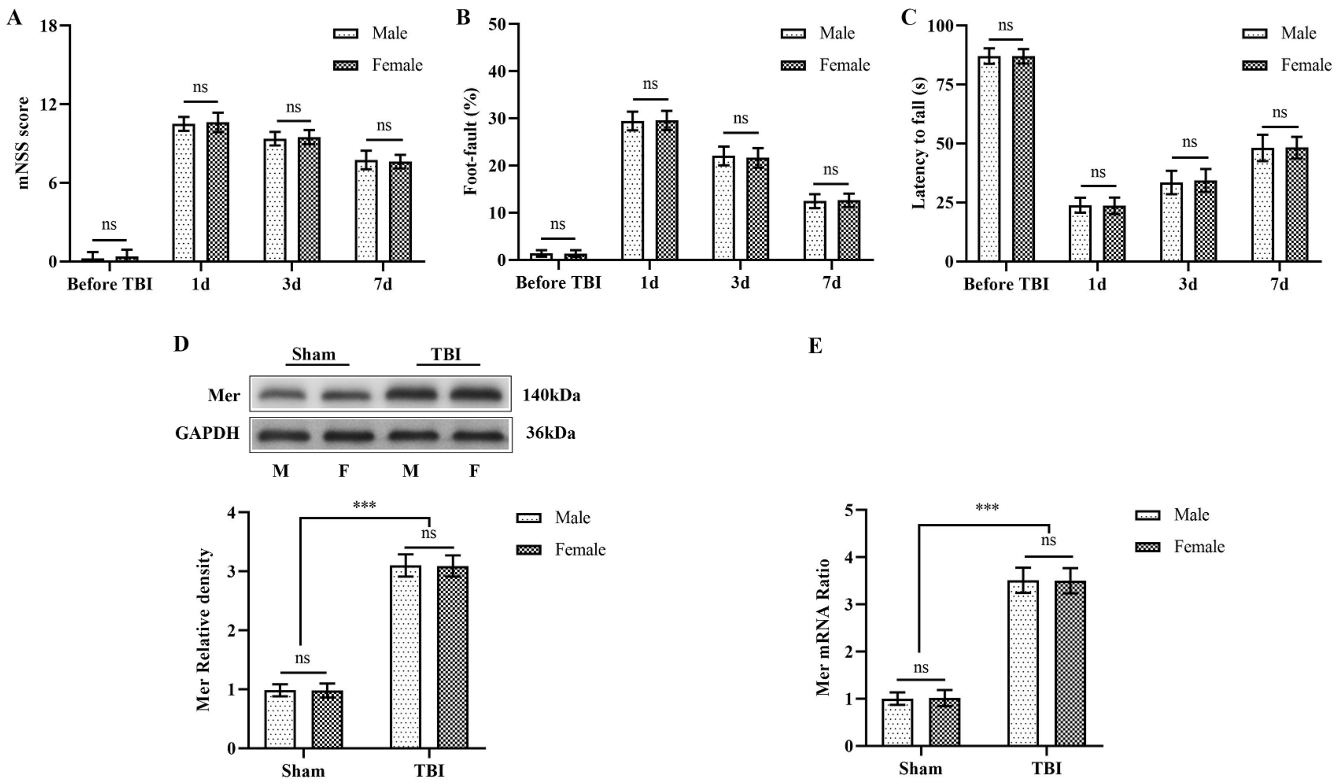

Supplementary Figure 4. (A) Modified neurological severity scores (mNSS), **(B)** foot-fault test, and **(C)** rotarod test were performed before and 1, 3, and 7 d after TBI. n = 8 mice per group. **(D)** Representative immunoblots and quantification showing the expression level of Mer protein in the injured cortex at 3 d after TBI or the equivalent area of the sham-operated brains. Data are expressed as fold change compared to sham-operated controls. n = 6 mice per group. M: male; F: female. **(E)** Quantitative RT-PCR was used to assess the mRNA expression level of Mer in the injured cortex at 3 d after TBI or the equivalent area of the sham-operated brains. Data are expressed as fold change compared to sham-operated controls. n = 6 mice per group. In **A-E**, data are presented as mean ± SD; ***, $p < 0.001$; ns, non-significant, $p > 0.05$. two-way ANOVA followed by Bonferroni's post-hoc tests.
