# Supplementary material for: Mer regulates microglial/macrophage M1/M2 polarization and alleviates neuroinflammation following traumatic brain injury
Source: J Neuroinflammation. 2021 Jan 5;18:2. doi: 10.1186/s12974-020-02041-7 (PMC7787000; doi:10.1186/s12974-020-02041-7)
Supplement: Supplementary file 5 — Additional file 5: Supplementary Figure 5. (A) Fluorescence intensity quantification of Mer expression in CD16/32- and CD206- positive cells in the impacted ipsilateral cortical area at 3 d after TBI. n = 6 per group. (B-G) Quantitative RT-PCR analysis showing mRNA expression of CD16 (B), CD32 (C), iNOS (D), CD206 (E), Arg-1 (F), IL-10 (G) in the injured cortex at 3 d post-TBI. GAPDH: loading control. Data are expressed as fold change compared to the sham group; n = 6 mice per group. In A, data are presented as Mean ± SD; ***, p < 0.001 by Student’s t-test. In B-G, data are presented as Mean ± SD; ***, p < 0.001. one-way ANOVA followed by Bonferroni’s post-hoc tests. [file 12974_2020_2041_MOESM5_ESM.pdf]

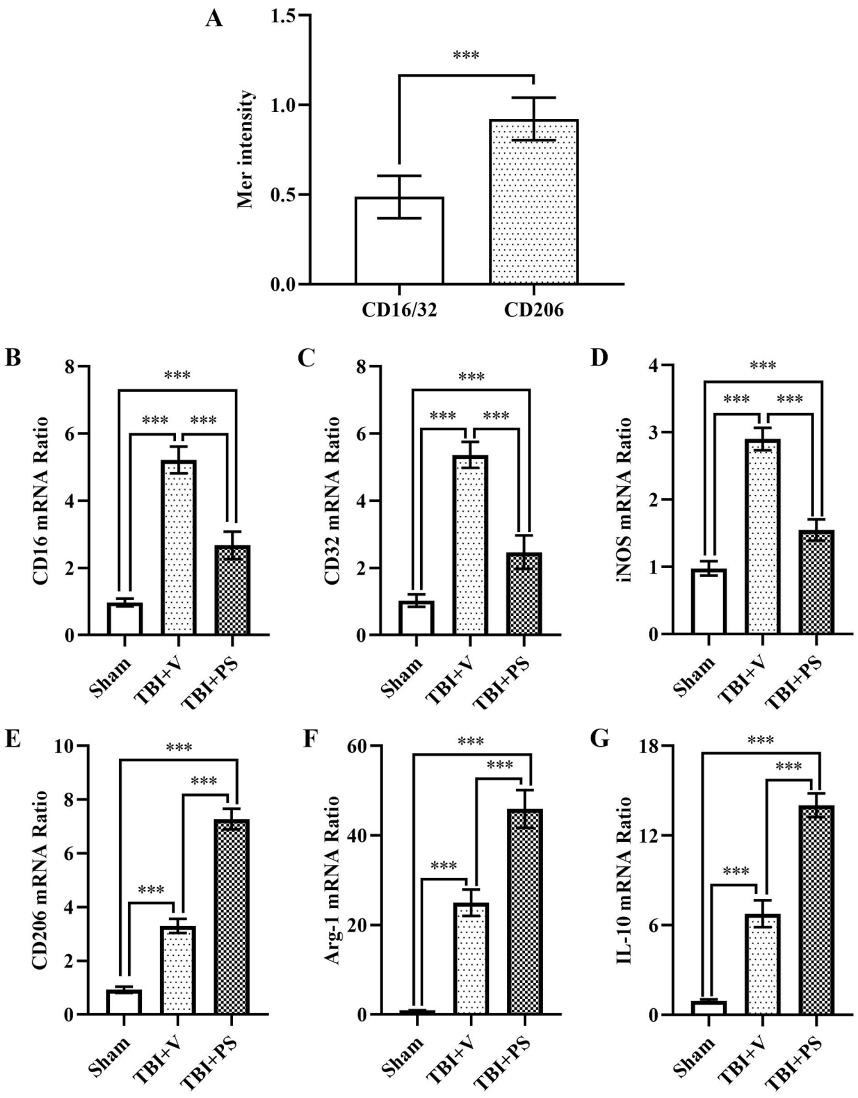

Supplementary Figure 5. (A) Fluorescence intensity quantification of Mer expression in CD16/32- and CD206-positive cells in the impacted ipsilateral cortical area at 3 d after TBI. n = 6 per group. **(B-G)** Quantitative RT-PCR analysis showing mRNA expression of CD16 **(B)**, CD32 **(C)**, iNOS **(D)**, CD206 **(E)**, Arg-1 **(F)**, IL-10 **(G)** in the injured cortex at 3 d post-TBI. GAPDH: loading control. Data are expressed as fold change compared to the sham group; n = 6 mice per group. In **A**, data are presented as Mean ± SD; ***, $p < 0.001$ by Student's t-test. In **B-G**, data are presented as Mean ± SD; ***, $p < 0.001$. one-way ANOVA followed by Bonferroni's post-hoc tests.
