# Supplementary material for: Mer regulates microglial/macrophage M1/M2 polarization and alleviates neuroinflammation following traumatic brain injury
Source: J Neuroinflammation. 2021 Jan 5;18:2. doi: 10.1186/s12974-020-02041-7 (PMC7787000; doi:10.1186/s12974-020-02041-7)
Supplement: Supplementary file 6 — Additional file 6: Supplementary Figure 6. (A) Cerebral edema was measured by brain water content. Quantification analysis showing TBI increased brain edema in the ipsilateral hemisphere on day 3 after TBI, which is significantly alleviated after PS treatment. n = 8 mice per group. (B) Representative images of Nissl staining in the ipsilateral cortex from the sham, TBI + Vehicle (TBI + V), TBI + protein S (TBI + PS) groups, respectively. (C) Quantification analysis showing TBI caused a significant decrease in the number of Nissl-positive cells in the ipsilateral cortex at 3 d post-TBI, and PS application significantly increased the number of Nissl-positive cells in the injured cortex after TBI. n = 6 mice per group. Scale bar = 20 μm. (D) Representative images of Fluoro-Jade B (FJB) staining in the ipsilateral cortex from the sham, TBI + V, TBI + PS groups, respectively. (E) Quantification analysis showing TBI caused a significant increase in the number of FJB-positive cells in the ipsilateral cortex at 3 d post-TBI, and PS application significantly decreased the number of FJB-positive cells in the injured cortex after TBI. n = 6 mice per group. Scale bar = 15 μm. In A, C, E, data are presented as Mean ± SD; **, p < 0.01; ***, p < 0.001. one-way ANOVA followed by Bonferroni’s post-hoc tests. [file 12974_2020_2041_MOESM6_ESM.pdf]

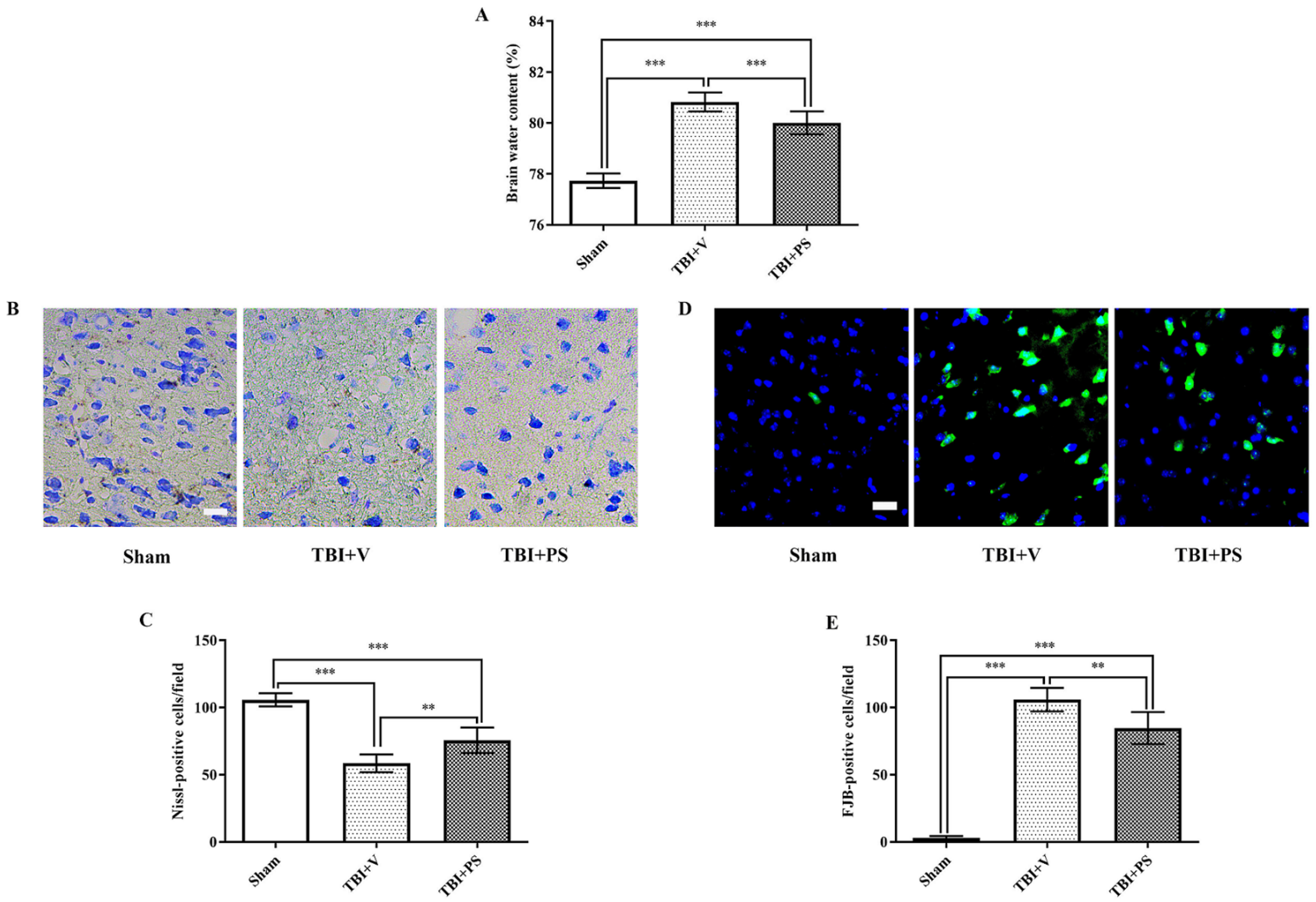

Supplementary Figure 6. (A) Cerebral edema was measured by brain water content. Quantification analysis showing TBI increased brain edema in the ipsilateral hemisphere on day 3 after TBI, which is significantly alleviated after PS treatment. n = 8 mice per group. **(B)** Representative images of Nissl staining in the ipsilateral cortex from the sham, TBI + Vehicle (TBI + V), TBI + protein S (TBI + PS) groups, respectively. **(C)** Quantification analysis showing TBI caused a significant decrease in the number of Nissl-positive cells in the ipsilateral cortex at 3 d post-TBI, and PS application significantly increased the number of Nissl-positive cells in the injured cortex after TBI. n = 6 mice per group. Scale bar = 20 μm. **(D)** Representative images of Fluoro-Jade B (FJB) staining in the ipsilateral cortex from the sham, TBI + V, TBI + PS groups, respectively. **(E)** Quantification analysis showing TBI caused a significant increase in the number of FJB-positive cells in the ipsilateral cortex at 3 d post-TBI, and PS application significantly decreased the number of FJB-positive cells in the injured cortex after TBI. n = 6 mice per group. Scale bar = 15 μm. In **A**, **C**, **E**, data are presented as Mean ± SD; **, $p < 0.01$; ***, $p < 0.001$. one-way ANOVA followed by Bonferroni's post-hoc tests.
